# Comparison of embryologist stress, somatization, and burnout reported by embryologists working in UK HFEA-licensed ART/IVF clinics and USA ART/IVF clinics

**DOI:** 10.1093/humrep/deae191

**Published:** 2024-08-28

**Authors:** Anar Murphy, Mark S Lapczynski, Glenn Proctor, Timothy R Glynn, Alice D Domar, Sofia Gameiro, Giles A Palmer, Michael G Collins

**Affiliations:** Department of Scientific Affairs, TMRW Life Sciences, New York, NY, USA; Department of Scientific Affairs, TMRW Life Sciences, New York, NY, USA; Conceptions Reproductive Associates of Colorado—IVI RMA, Littleton, CO, USA; Dudley Associates Healthcare Marketing and Communications, Maplewood, NJ, USA; Inception Fertility, Houston, TX, USA; School of Psychology, Cardiff University, Cardiff, UK; International IVF Initiative, New York, NY, USA; Institute of Life, Athens, Greece; IVF 2.0 Ltd, London, UK; Department of Scientific Affairs, TMRW Life Sciences, New York, NY, USA

**Keywords:** stress, somatization, burnout, anxiety, ART/IVF, work conditions

## Abstract

**STUDY QUESTION:**

What is the prevalence of occupational stress, somatization, and burnout reported by UK and US, embryologists and the impact of work conditions on these well-being outcomes?

**SUMMARY ANSWER:**

Surveyed UK and US embryologists reported moderate perceived stress, low somatic symptom severity, high levels of burnout, and overall stressful work conditions, but with differences that could be due to country-specific occupational and employment characteristics.

**WHAT IS KNOWN ALREADY?:**

Spanish, UK, US, and international surveys have identified high levels of occupational stress, somatization, burnout, and occupational health issues among embryologists. These issues have been attributed to embryologists’ occupational challenges and work conditions.

**STUDY DESIGN, SIZE, DURATION:**

A cross-sectional web-based survey was sent to 253 embryologists working in UK ART/IVF clinics and 487 embryologists working in US ART/IVF clinics.

**PARTICIPANTS/MATERIALS, SETTING, METHODS:**

Participants self-reported their stress levels, somatization, burnout, and work conditions. Proportions across the Perceived Stress Scale (PSS), Patient Health Questionnaire (PHQ-15), Maslach Burnout Inventory-General Survey (MBI-GS), a single-item work unit grade (A–F), and customized occupational and sociodemographic questionnaires were calculated using descriptive statistics. Welch’s *t*-test was utilized to compare PSS and PHQ-15 scores between groups. Risk ratios were calculated using log-binomial regression for all models except for levels of anxiety related to performing cryostorage tasks, for which Poisson models were used.

**MAIN RESULTS AND THE ROLE OF CHANCE:**

In total, 50.6% (128) of the embryologists in the UK and 50.1% (244) in the US completed the survey. Both groups self-reported moderate PSS and low PHQ-15 scores, although fewer UK embryologists scored high on the MBI cynicism dimension than their US colleagues (43% UK vs 60% US embryologists, *P *<* *0.05). The UK and US embryologists did not differ on the MBI exhaustion dimension with both scoring high for exhaustion (59% UK vs 62% US). Although 81% and 80% of UK and US embryologists, respectively, reported working overtime, more embryologists in the UK reported being adequately compensated. Increasing levels of anxiety-related to cryostorage showed a dose-dependent increased risk of burnout on at least two MBI-GS dimensions only in the UK group, and, a dose-dependent likelihood of higher PSS and PHQ-15 scores in both groups.

**LIMITATIONS, REASONS FOR CAUTION:**

Since the two groups were surveyed 9 months apart and were self-reporting, the study is limited by the differences in responsibilities, scheduling, and workload specific to the time of year.

**WIDER IMPLICATIONS OF THE FINDINGS:**

Work-related health issues and occupational challenges shared by UK and US embryologists could be addressed by organizational enhancements and technology. Lower levels of stress and burnout among UK embryologists might be due to the HFEA-provided structure/certainty.

**STUDY FUNDING/COMPETING INTEREST(S):**

This study was supported without any external funding by TMRW Life Sciences Inc., which is developing and commercializing an automated platform for embryology. M.G.C. and M.S.L. are full-time employees and stockholders/shareholders with TMRW Life Sciences, and A.M. of Novavax, Inc. was an employee of TMRW Life Sciences. G.P. is a consultant for TMRW Life Sciences. The remaining authors declare no conflict of interest.

**TRIAL REGISTRATION NUMBER:**

NCT05326802; NCT05708963.

## Introduction

Clinical embryologists are exacting, highly trained members of ART/IVF clinics who carry out the diagnosis and treatment of infertility by manipulating and preserving fresh and frozen reproductive specimens, testing them for genetic and infectious diseases, maintaining and managing embryology laboratories and cryostorage facilities, and, ultimately, helping patients fulfill their family-building goals (Practice Committee of the American Society for Reproductive Medicine, Practice Committee of the Society for Assisted Reproductive Technology, and Practice Committee of the Society of Reproductive Biologists and Technologists, 2014, 2021). Work-related stress, burnout, and anxiety are known to decrease work performance (Ruotsalainen *et al.*, 2014). The negative effects of work conditions and work-related problems on the emotional and physical well-being of workers became a subject of scientific interest in the mid-1970s (Freudenberger, 1974; Punnett and Wegman, 2004), culminating in the development of the Maslach Burnout Inventory (MBI; Maslach and Jackson, 1981). Combined with other reliable tools measuring work-related stress, somatization, fatigue, and other psychological and physical disorders among workers employed in various fields, the MBI has become a gold standard for measuring burnout. However, more work needs to be done to define burnout (Maslach *et al.*, 2001; Guseva Canu *et al.*, 2021; Schaufeli, 2021, 2023), and each field needs a customized toolkit to assess occupational challenges and health issues specific to its workers (Frone and Tidwell, 2015).

The first study of occupational health issues among embryologists was conducted in Spain via an online health questionnaire distributed among members of the Spanish Association of Clinical Embryologists (López-Lería *et al.*, 2014). Several other groups have studied occupational health issues among embryologists in the UK (Priddle *et al.*, 2022; Murphy *et al.*, 2023a, 2023d), the US (Murphy *et al.*, 2022a, 2023a, 2023b, 2023c), and an international cohort of embryologists from 85 countries (Palmer *et al.*, 2022) using different survey toolkits.

High levels of occupational stress, somatization, and burnout in addition to other occupational health issues among embryologists have been reported in several studies (López-Lería *et al.*, 2014; Collins *et al.*, 2022; Murphy *et al.*, 2022a, 2023a, 2023b, 2023c, 2023d; Palmer *et al.*, 2022; Priddle *et al.*, 2022) and attributed to stressful work conditions in the embryology laboratory. Stressors include the lack of room for error since mishandling reproductive specimens can lead to devastating consequences for the patient(s) and legal implications for the providers (Letterie, 2017; Moutos *et al.*, 2019; Letterie and Fox, 2020; Murphy *et al.*, 2022b; Applebaum *et al.*, 2023; Klipstein and Daar, 2023). Additionally, embryologists face a heavy workload with long hours and frequent holiday/weekend work (Murphy *et al.*, 2022a, 2023a), performing repetitive and fatiguing tasks using manual procedures (López-Lería *et al.*, 2014; Collins *et al.*, 2022; Priddle *et al.*, 2022), and an overwhelming amount of paperwork (López-Lería *et al.*, 2014; Go, 2015; Murphy *et al.*, 2022a, 2023a, 2023b, 2023c, 2023d).

However, there is still a significant knowledge gap among clinical laboratory professionals (Thompson *et al.*, 2003; Lorusso *et al.*, 2007; Fritzsche *et al.*, 2012). This includes embryologists, and the occupational challenges and health issues arising from their work (López-Lería *et al.*, 2014; Campbell *et al.*, 2022; Collins *et al.*, 2022; Murphy *et al.*, 2022a, 2023a, 2023b, 2023c, 2023d; Palmer *et al.*, 2022; Priddle *et al.*, 2022), while the same issues among other healthcare professionals have been the subject of numerous studies (Boivin *et al.*, 2017; Dyrbye *et al.*, 2018, 2019; Tawfik *et al.*, 2018; West *et al.*, 2018; Caruso *et al.*, 2019; Trockel *et al.*, 2020; Brady *et al.*, 2021, 2022; Shanafelt, 2021; Rowe *et al.*, 2022; Shanafelt *et al.*, 2022, 2023b, 2023c; Longo *et al.*, 2023; Makowski *et al.*, 2023; Marchalik and Shanafelt, 2023; Sinsky *et al.*, 2023). Moreover, despite their indispensable role in fertility treatments and patient outcomes, embryologists are not generally considered healthcare professionals by regulatory bodies, and their occupational concerns and needs are often overlooked by their employers (Kovačič *et al.*, 2015; ESHRE Working Group on Embryologist Training Analysis *et al.*, 2023). The scarcity of real-life evidence and the lack of understanding about embryologists’ occupational challenges by their employers may contribute to stress, somatization, and burnout, which can have short- and long-term negative consequences for embryologists, their work, and the clinics (Maslach *et al.*, 2001; Guseva Canu *et al.*, 2021; Schaufeli, 2021, 2023).

Compounding this issue, embryologists in different countries and different states within the US may operate under different legal and regulatory conditions, which may result in varied access to resources and can either alleviate or exacerbate their occupational challenges and health issues. Therefore, studies carried out in a single country might be highly specific to that country, and international studies that cover multiple countries might not account for the local characteristics of each participant’s work conditions.

The present study seeks to compare stress, somatization, and burnout among embryologists in the UK and the US. Recently, cryostorage expansion has increased the demands on embryologists (Alikani *et al.*, 2014; Go, 2015, 2019; Alikani, 2018; Alikani and Parmegiani, 2018). The current workflow and organizational characteristics of the embryology laboratories are investigated to determine if there is an association with embryologists’ physical and psychological health. Additionally, the relationship between anxiety associated with specific workflows, such as cryostorage, on embryologists’ stress, somatization, and burnout is determined. The results of this study will identify necessary areas of improvement in the embryology laboratory that could increase patient care by alleviating embryologist stress, somatization, and burnout.

## Materials and methods

### Study design and participants

This study used data from the Embryologist Fatigue Study (EFS), carried out as a cross-sectional web-based survey emailed to 253 embryologists working at HFEA-licensed UK (Murphy *et al.*, 2023d) ART/IVF public and private clinics and 487 embryologists working at College of American Pathologists (CAP) accredited and/or The Joint Commission (TJC) certified or non-accredited US clinics (Murphy *et al.*, 2022a). Responses were collected for two weeks in January 2023 and April 2022, respectively. This data was collected using a customized survey toolkit where respondents were asked to self-report their stress levels, physical health status, burnout, and work conditions that they perceived as related to their occupation as an embryologist. The survey toolkit used validated standardized psychological and somatic symptom assessment tools and customized sociodemographic and occupational questionnaires. The email list(s) were compiled from the authors’ known contacts in the field of Reproductive Health, and by actively gathering contacts in the years preceding the study by other members of the authors’ organizations. The list is periodically updated to maintain as accurate contact information as possible.

All survey participants were required to be practicing embryologists at different stages of their careers and to complete all parts of the survey. No other exclusion/inclusion criteria were applied to the study groups.

The studies were registered on ClinicalTrials.gov (US EFS: NCT05326802; and UK EFS: NCT05708963).

### Sample characteristics

Sociodemographic information regarding age, gender, education, years of experience, and other key descriptive characteristics of the survey participants was collected using a customized sociodemographic questionnaire.

### Symptoms of stress, somatization, and burnout

Embryologists self-reported perceived stress using the standardized validated Perceived Stress Scale (PSS)-10 (Cohen *et al.*, 1983). Items are scored on a 5-point scale from 0 (‘never’) to 4 (‘often’), with some inversion. Total scores of 0–13 are considered low perceived stress, 14–26 are moderate stress, and 27–40 are high stress.

Somatic symptom severity was measured using the standardized validated 15-question Patient Health Questionnaire (PHQ-15) (Kroenke *et al.*, 2002). Items are scored on a 3-point scale of 0 (‘not bothered at all’), 1 (‘bothered a little’), and 2 (‘bothered a lot’). Somatization is categorized from total scores as minimal (0–4), low (5–9), medium (10–14), and high (15–30).

Burnout was assessed using the MBI-GS (Mind Garden™, Menlo Park, CA), and responses were scored according to the MBI Manual 3rd edition (Maslach *et al.*, 1996), as described in López-Lería *et al.* (2014). The MBI-GS assesses burnout on three dimensions: Exhaustion, Cynicism, and Professional Efficacy. Results on these dimensions are categorized as low, medium, or high burnout (Schaufeli *et al.*, 1996; López-Lería *et al.*, 2014; see Results below and Corresponding Supplementary Figures). High burnout was defined as having burnout scores in the third tertile in at least two of the three burnout dimensions.

### Working conditions

Embryologists self-reported their satisfaction with their workplace culture, careers and future career outlook, job fulfillment, scheduling and overtime concerns, and burdens associated with technical tasks specific to their occupation in a customized occupational questionnaire. A ‘Technology & The Lab’ section consisted of five items (e.g. ‘Do any of the current cryostorage processes cause you anxiety?’), ‘Career Outlook’ eight items (e.g. ‘How likely are you to recommend a career in embryology to a new college graduate or a person looking to change career paths?’), ‘Physical and Mental Stress Levels’ eight items (e.g. ‘How often do you experience stress when depositing and retrieving oocytes and embryos from cryostorage?’), and the ‘Scheduling/Staffing & Time Off’ nine items (e.g. ‘How many hours of overtime do you typically work per week? Is overtime voluntary or mandatory?’). Several items included questions that required ‘Yes/No’, ‘Yes/No/Maybe/I don’t know’, or ‘Very Often/Often/Rarely/Never’ pre-formulated multiple choice, and open-ended answers. Items that asked respondents to score their career satisfaction and outlook, job fulfillment, and stress levels associated with performing cryopreservation tasks, such as freezing/thawing reproductive specimens and handling tanks, were scored on a 10-point Likert-type scale ranging from 1 (‘lowest/never/least’) to 10 (‘highest/always/most’). Respondents were asked to rank their levels of anxiety related to performing cryostorage tasks, from freezing/thawing reproductive specimens to handling cryotanks and managing cryoinventory, on a 5-point Likert-type scale ranging from 1 (‘No Anxiety) to 5 (‘Constant Anxiety’).

### Statistics

Descriptive statistics for stress, somatization, burnout, and sociodemographic characteristics were presented as the mean ± SD or as count and proportion. Mean differences between groups within each study population were compared using Welch’s *t*-test. Two independent population proportions were compared using the *Z*-test.

Log-binomial models were used to estimate the unadjusted and adjusted risk ratio (RR) and 95% CI of having high levels of burnout associated with various characteristics of the work environment. Linear models were used to estimate the adjusted change and 95% CI in PSS and PHQ-15 scores associated with various characteristics of the work environment. All models were adjusted for years of experience as an embryologist, full vs part-time work, and having vs not having a doctoral degree, as covariates. All analyses were conducted with SAS^®^ 9.4 Software (SAS Institute, Cary, NC).

### Ethics

The US survey was reviewed by an IRB (Pro00062375, Center for IRB Intelligence (CIRBI) Platform, Advarra, Columbia, MD). The determination was that the study did not require IRB oversight. The UK survey was determined to be exempt from an NHS Research Ethics Committee (REC) review by using the ‘Do I need NHS REC review?’ tool (NHS Health Research Authority, https://www.hra-decisiontools.org.uk/ethics/index.html).

The participants in both groups provided their informed consent prior to completing the survey. Participants in the US and the UK received an Amazon gift card with a value of $25 and £25, respectively, upon completion of the entire survey.

## Results

### Sample description

**Figure 1.**
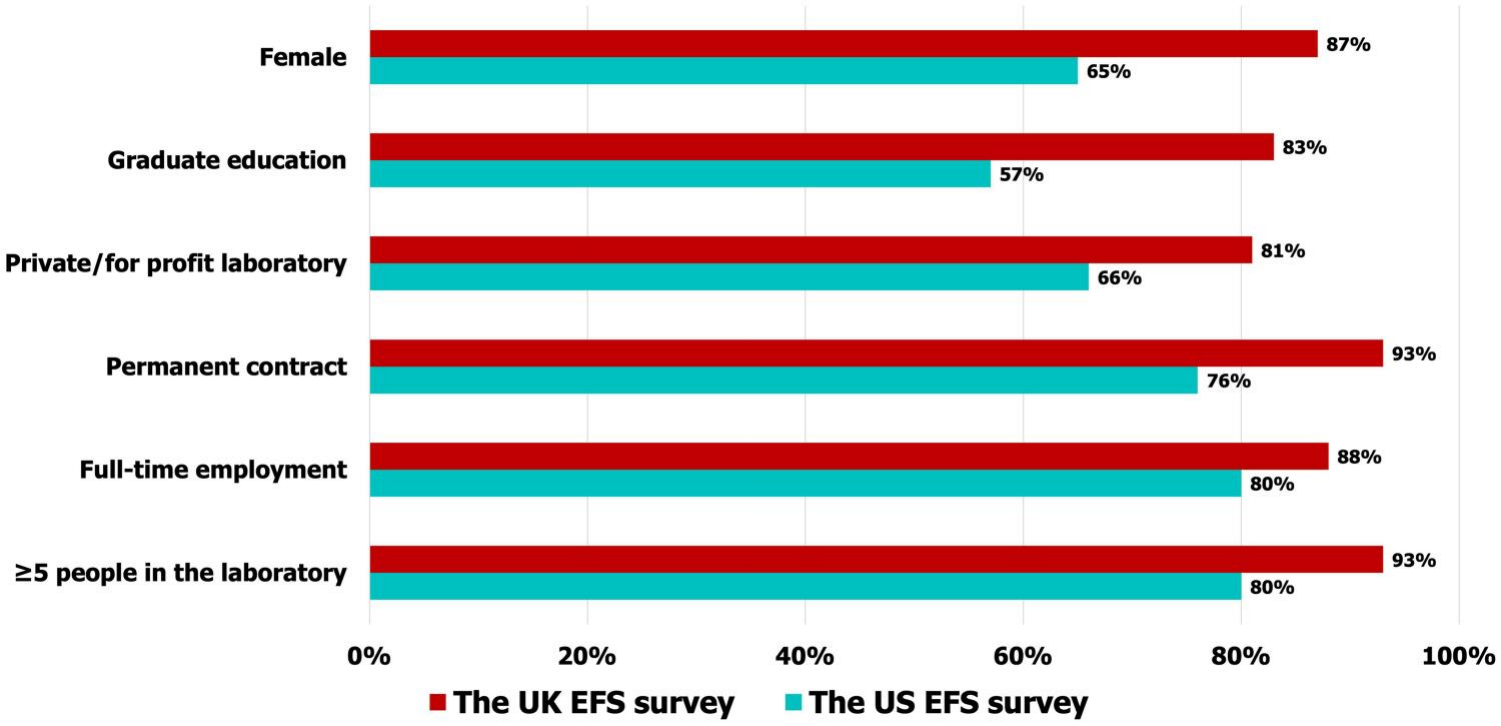
**Sociodemographic characteristics and employment characteristics of embryologists working in the HFEA-licensed UK clinics and CAP-accredited and/or TJC-certified or non-accredited US public and private ART/IVF clinics.** ART/IVF, assisted reproductive technology/*in vitro* fertilization; CAP, College of American Pathologists; HFEA, Human Fertilisation & Embryology Authority; TJC, The Joint Commission.

In total, 50.6% (128) of the embryologists in the UK and 50.1% (244) in the US completed the survey. The UK survey participants were, on average, 34.4 years old compared to their US colleagues whose mean age was 40.4 years, and although not statistically significant, more UK participants were female (87% UK and 65% US). Figure 1 compares the education and key employment characteristics of UK and US participants. (For sociodemographic data, see Supplementary Fig. S1; for PSS and PHQ-15 values, see Supplementary Figs S2 (UK) and S3 (US)).

### Self-reported stress, somatization, and burnout

There was no difference between the two countries for the self-reported moderate PSS stress scores, but embryologists in the UK scored slightly lower on PHQ-15 than their colleagues in the US (*P *=* *0.04). For a detailed comparison of responses to each question in PSS-10, see Supplementary Figs S4 (UK vs US), S5 (UK), and S6 (US), and in PHQ-15, Supplementary Figs S7 (UK vs US), S8 (UK), and S9 (US).

The MBI showed high levels of burnout on the exhaustion dimension in both groups (59% UK vs 62% US embryologists, *P *=* *0.27). Fewer UK embryologists scored high on the cynicism (43% UK vs 60% US embryologists, *P *≤* *0.05) and professional efficacy (15% UK vs 28% US embryologists, *P *≤* *0.05) MBI dimensions than their US colleagues (Supplementary Figs S10 (UK vs US), S11 (UK), and S12 (US) and Supplementary Data File S1).

### Work conditions

**Figure 2.**
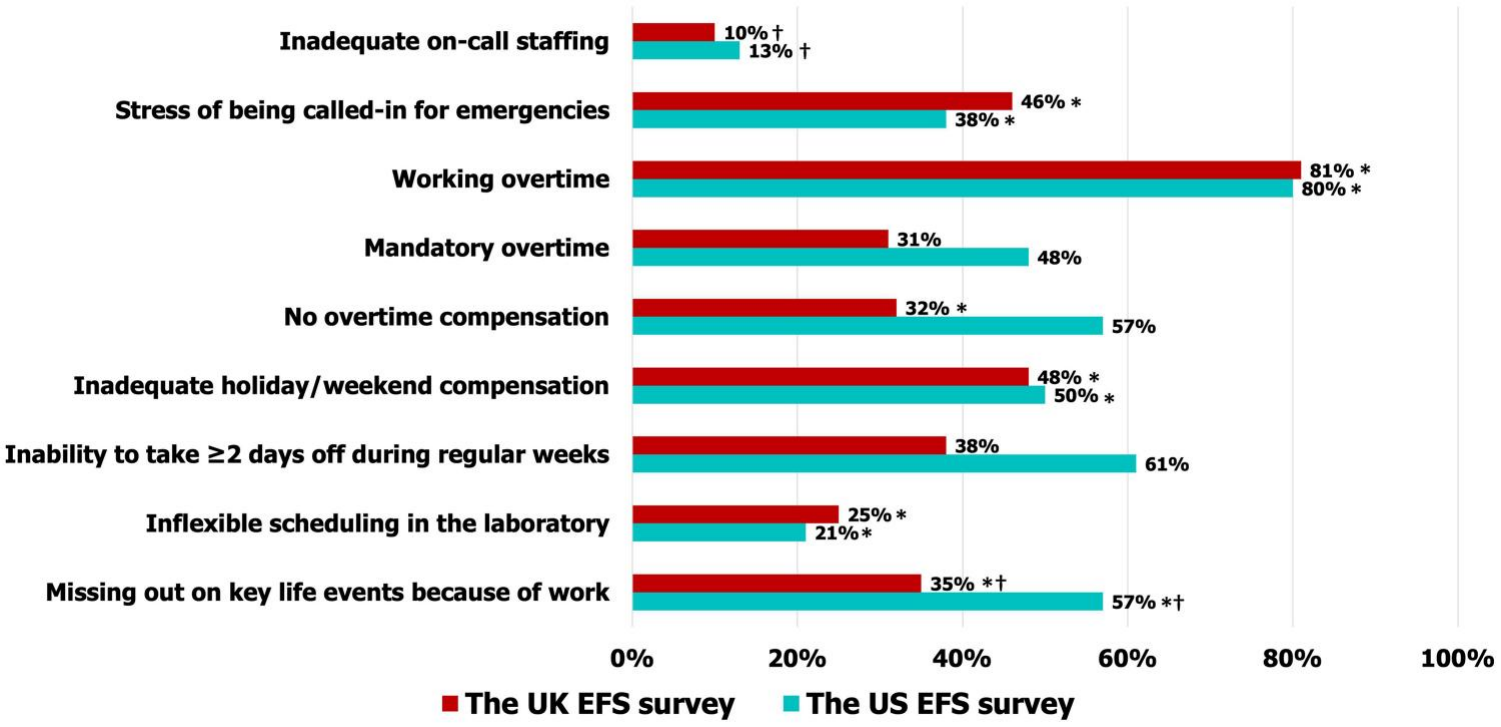
**Employment characteristics and work conditions in the embryology laboratory reported by participants of the UK and US surveys.** *, a statistically significant increased risk of burnout in at least two MBI domains associated with employment characteristics/work conditions; †, a statistically significant increase in PSS and PHQ-15 associated with employment characteristics/work conditions. MBI, Maslach Burnout Inventory; PHQ-15, a 15-question Patient Health Questionnaire; PSS, Perceived Stress Scale.

Of embryologists in the UK survey, 83% had a graduate degree compared to 57% of their colleagues in the US survey, and 93% of UK embryologists worked on a permanent contract basis compared with 76% of their colleagues in the US. Of the embryologists in the UK survey, 88% were employed on a full-time basis compared to 80% of their US colleagues, and 6% of the UK embryologists worked for corporate employers vs 14% of their US colleagues. Furthermore, 93% of the surveyed UK embryologists worked in laboratories with ≥5 persons (considered as adequately staffed; Alikani *et al.*, 2014; Veiga *et al.*, 2022) compared to 80% of the US embryologists (Fig. 1). When comparing organizational characteristics, although a similar number of participants in both groups reported working overtime (81% of UK and 80% of US embryologists, *P *=* *0.63), a smaller proportion of UK embryologists reported being not compensated for overtime (*P *≤* *0.05), not being able to take off 2 or more consecutive days during regular weeks (*P *≤* *0.05), and missing out on key life events due to work (*P *≤* *0.05) compared to their US colleagues (Fig. 2; Supplementary Figs S13 and S14 (UK) and S15 and S16 (US)).

### Association of work conditions and anxiety with stress, somatization, and burnout

In both the UK and the US results/studies, there were significantly greater PSS stress scores associated with inadequate staffing (4.66, 95% CI: 2.42–6.90 and 3.09, 95% CI: 1.47–4.72, respectively) and ever-missing life events due to work (4.08, 95% CI: 1.79–6.38, and 3.71, 95% CI: 2.02–5.41, respectively). In the US results, higher PSS scores were also significantly associated with anxiety related to being on-call (2.51, 95% CI: 1.06–3.95) and having inflexible scheduling (2.93, 95% CI: 1.55–4.30) (Supplementary Table S1). Regarding PHQ-15 scores, in both groups, significantly higher PHQ-15 scores were associated with inadequate staffing (1.96, 95% CI: 0.17–3.76, and 2.34, 95% CI: 0.83–3.85, respectively) and missing out on key life events due to work (2.85, 95% CI: 1.08–4.61, and 3.36, 95% CI: 1.80–4.92, respectively). In the US, inflexible scheduling was also associated with higher PHQ-15 scores (1.59, 95% CI: 0.30–2.88) (Supplementary Table S1).

In both the UK and the US studies, the risk of having high levels of burnout in at least two dimensions was significantly associated with anxiety related to being on-call (aRR: 1.62, 95% CI: 1.13–2.31, and aRR: 1.32, 95% CI: 1.13–1.54, respectively), inflexible scheduling (aRR: 1.49, 95% CI: 1.09–2.04, and aRR: 1.21, 95% CI: 1.02–1.43, respectively), and missing out on key life events due to work (aRR: 1.97, 95% CI: 1.36–2.85, and aRR: 1.58, 95% CI: 1.17–2.14, respectively). In the UK results, the risk of burnout was significantly associated with working overtime (aRR: 1.82, 95% CI: 1.01–3.29) and inadequate evening and weekend compensation (aRR: 1.64, 95% CI: 1.04–2.61). In the US results, the risk of burnout was significantly associated with inadequate on-call staffing (aRR: 1.63, 95% CI: 1.22–2.17) and mandatory overtime (aRR: 1.22, 95% CI: 1.02–1.47) (Supplementary Table S2).

### Effects of specific cryostorage-related anxiety levels on perceived stress, somatization, and burnout

Half of the respondents in both study groups reported anxiety related to cryostorage (data not shown). While there was a difference in the proportions of UK and US embryologists reporting constant, high, and moderate levels of cryostorage-related anxiety, only the difference in the proportions of those reporting constant anxiety was statistically significant (*P *≤* *0.05; Supplementary Figs S17 (UK) and S18 (US) and Supplementary Data File S1). Only the UK survey respondents self-reported a dose-dependent anxiety-related clinically significant increased risk of burnout on at least two MBI-GS dimensions (Table 1).

**Table 1.** Effects of cryostorage-related anxiety levels on burnout.

Cryostorage-related anxiety levels	The UK survey		The US survey	
	Unadjusted RR (95% CI)	Adjusted* **aRR (95% CI)**	Unadjusted RR (95% CI)	Adjusted* **aRR (95% CI)**
None	Ref	Ref	Ref	Ref
Mild	1.23 (0.53–2.86)	1.19 (0.51–2.82)	1.69 (0.99–2.89)	1.70 (1.02–2.83)
Moderate	1.51 (0.64–3.58)	1.38 (0.56–3.38)	**2.10 (1.24–3.54)**	2.09 (1.26–3.46)
High	2.22 (0.93–5.28)	2.13 (0.87–5.22)	1.33 (0.75–2.36)	1.57 (0.90–2.75)
Constant	2.57 (0.67–9.94)	2.64 (0.68–10.3)	1.51 (0.81–2.80)	1.69 (0.95–3.02)

Cryostorage-related anxiety levels showed a clinically significant dose-dependent increased risk of burnout on at least two MBI-GS dimensions in the UK survey participants.

* Adjusted for years as an embryologist, full-time vs part-time/per-diem working status, and doctorate-level education. Bold denotes a statistically significant dose-dependent effect of cryostorage-related anxiety on burnout.

PSS scores also significantly increased with the amount of anxiety related to cryostorage; larger increases in scores were observed in the UK results (range: 3.89 [95% CI: 0.85–6.94] for mild anxiety to 18.9 [95% CI: 12.2–25.7] for constant anxiety) compared to the US results (range: 2.49 [95% CI: 0.02–4.97] for mild anxiety to 6.44 [95% CI: 3.28–9.60] for constant anxiety). In both the UK and US groups, cryostorage-related anxiety was associated with higher PHQ-15 scores (UK range: 3.25 [95% CI: 0.57–5.92] for mild anxiety to 7.76 [95% CI: 1.83–13.7] for constant anxiety; US range: 1.96 [95% CI: −0.34 to 4.25] for mild anxiety to 5.17 [95% CI: 2.24–8.11] for constant anxiety, respectively). However, the association with mild anxiety did not reach statistical significance for the US respondents (Table 2).

**Table 2.** Effects of cryostorage-related anxiety on perceived stress and somatic symptom severity.

Cryostorage-related anxiety levels	The UK survey		The US survey	
	PSS change (95% CI)	PHQ-15 change (95% CI)	PSS change (95% CI)	PHQ-15 change (95% CI)
None	Ref	Ref	Ref	Ref
Mild	**3.89 (0.85–6.94)**	**3.25 (0.57–5.92)**	**2.49 (0.02–4.97)**	1.96 (−0.34 to 4.25)
Moderate	**7.60 (4.29–10.9)**	**5.25 (2.35–8.16)**	**5.73 (3.23–8.26)**	**4.02 (1.70–6.34)**
High	**10.7 (7.15–14.2)**	**5.48 (2.37–8.59)**	**5.24 (2.57–7.91)**	**5.43 (2.96–7.91)**
Constant	**18.9 (12.2–25.7)**	**7.76 (1.83–13.7)**	**6.44 (3.28–9.60)**	**5.17 (2.24–8.11)**

Change and 95% CI in Perceived Stress Scale (PSS) and Patient Health Questionnaire (PHQ)-15 scores adjusted for the following variables: years as an embryologist, full-time vs part-time/per-diem working status, and doctorate-level education. Bold denotes a statistically significant dose-dependent effect of cryostorage-related anxiety on PSS and PHQ-15 changes.

## Discussion

### Interpretation of the survey results

Findings from this study confirm that embryologists are vulnerable to stress and burnout when working in HFEA-licensed UK ART/IVF and US private and public ART/IVF clinics. There are specific working conditions that seem to contribute to the risk of burnout and worse well-being.

By combining validated, standardized psychological and somatic symptom assessment tools and customized occupational and sociodemographic questionnaires, and asking embryologists about work conditions, job fulfillment and career satisfaction, stress, somatization, anxiety, burnout factors, and other occupational aspects that are exclusive to embryologists, it was possible to narrow down their emotional and physical health issues.

The standardized questionnaires showed that embryologists in both groups experienced moderate levels of self-perceived stress and low somatic symptom severity. Working at the bench and computer stations and performing manual cryopreservation and cryostorage procedures involve prolonged periods of sitting, standing, reaching out, bending, and lifting heavy objects, which, in combination with the scheduling specifics in ART/IVF laboratories that require overtime and weekend/holiday work, create a stressful and fatiguing working environment (Collins *et al.*, 2022; Murphy *et al.*, 2022a, 2023a, 2023b, 2023c, 2023d; Palmer *et al.*, 2022; Priddle *et al.*, 2022) for all embryologists, regardless of the country of their employment. There were differences in responses to individual questions in PSS and PHQ-15 questionnaires between the two groups, which could be due to differences in both work and national cultures, lifestyles, overall health, diets, and other factors specific to each country. However, the most prevalent somatic symptoms in both groups were back pain, feeling tired or low energy, and trouble sleeping, consistent with other studies on embryologists’ physical well-being (Priddle *et al.*, 2022) and fatiguing working conditions (Collins *et al.*, 2022).

When comparing MBI dimensions, both groups reported similar levels of burnout on the exhaustion dimension. However, embryologists working in UK clinics reported lower levels of burnout on cynicism and professional efficacy dimensions than their colleagues in the US, which could also be due to cultural differences between the two groups and strict HFEA oversight, and, therefore, embryologists working in UK clinics may feel they have more accountability and security in their jobs.

Stressful employment conditions in both groups, such as working overtime, lack of compensation for overtime and weekend/holiday work, inadequate staffing, being called in for emergencies, and missing out on key life events due to work, are more likely to be reflected in high levels of burnout. Additionally, inadequate staffing and missing out on key life events due to work are more likely to trigger higher levels of stress and somatization. These findings demonstrate the equal importance of a balanced and well-compensated work environment and life-work balance for embryologists working in both UK and US ART/IVF clinics.

The stronger dose dependence between burnout, perceived stress and somatic symptom severity, and the levels of cryostorage-related anxiety in embryologists working in UK clinics may be due to a structured regulatory environment and rigid reporting requirements implemented by HFEA, the statutory public body. Specifically, all UK ART/IVF clinics are licensed and regulated by HFEA, and they are legally required to report data on all aspects of their operations, including incident rates ranked by severity levels. Meanwhile, US ART/IVF clinics only report success rates and percentages of individual procedures to the CDC and SART, and laws pertaining to fertility procedures, specimen safety, and patient rights vary by state. Hence, handling frozen reproductive specimens and liquid nitrogen tanks may be more emotionally and physically straining to embryologists working in UK clinics because higher rates of IVF incidents may negatively impact their standing with HFEA. US colleagues do not carry the same legal responsibilities. In the US, most IVF incidents are handled at the clinic level according to the local state laws and are often settled out of court. Medicolegal experts can only estimate the rates of IVF incidents in the US based on the sparsely available public information, and their actual number is unknown (Moutos *et al.*, 2019; Letterie and Fox, 2020; Murphy *et al.*, 2022b; Applebaum *et al.*, 2023).

Regardless of embryologists’ country-specific organizational and employment characteristics, the stressful work conditions and occupational challenges reported by both study groups can have a similar negative impact on embryologists’ well-being, by increasing levels of occupational stress, somatization, and burnout. Those issues, in turn, may adversely affect the quality of embryologists’ work but can be addressed by organizational enhancements and common technology improvements.

### Strengths and limitations

This study is the first to compare the prevalence of stress, somatization, and burnout among embryologists in the UK and the US, the current workflow and organizational characteristics specific to each country, and how these differences affect embryologists’ physical and emotional health. The main limitation of this study is the self-reporting nature of the data collection via online surveys of a contact list of embryologists. The survey responses were collected from the two groups 9 months apart, which may affect their responses due to differences in the responsibilities, scheduling, and workload specific to the time of the year. The US survey was sent out in April 2022, shortly after 2 years of lockdowns, restrictions on elective medical procedures, and resulting scheduling uncertainties of the COVID-19 pandemic, all of which may have contributed to higher levels of stress, somatization, and burnout than in the UK where the participants completed the survey when those restrictions had been substantially lifted. Since the survey did not ask embryologists questions about the effects of the COVID-19 pandemic on their well-being during and after lockdowns, one can only speculate about this shortcoming. Therefore, it merits further studies similar to Shanafelt *et al.* (2023a) and Sinsky *et al.* (2023) that evaluated how the COVID-19 pandemic affected physicians’ well-being and desire to continue practicing medicine.

The average age of respondents in the UK survey was lower than in the US survey, suggesting that the US respondents were more likely to hold higher-level supervisory/managerial positions in their laboratories, and, therefore, their daily routine may involve more managerial and administrative tasks, as opposed to benchwork and cryopreservation/cryostorage tasks performed by younger colleagues.

### Future studies

Differences between their occupational challenges merit future studies targeting early- to mid-career embryologists in non-supervisory positions to identify and address occupational challenges and health issues specific to them. We could also directly compare work conditions and occupational health issues among embryologists with doctors and nurses working in ART/IVF clinics to identify challenges common to the ART/IVF field and those specific to working in the embryology laboratory.

## Supplementary Material

deae191_Supplementary_Data_File_S1

deae191_Supplementary_Figure_S1

deae191_Supplementary_Figure_S2

deae191_Supplementary_Figure_S3

deae191_Supplementary_Figure_S4

deae191_Supplementary_Figure_S5

deae191_Supplementary_Figure_S6

deae191_Supplementary_Figure_S7

deae191_Supplementary_Figure_S8

deae191_Supplementary_Figure_S9

deae191_Supplementary_Figure_S10

deae191_Supplementary_Figure_S11

deae191_Supplementary_Figure_S12

deae191_Supplementary_Figure_S13

deae191_Supplementary_Figure_S14

deae191_Supplementary_Figure_S15

deae191_Supplementary_Figure_S16

deae191_Supplementary_Figure_S17

deae191_Supplementary_Figure_S18

deae191_Supplementary_Table_S1

deae191_Supplementary_Table_S2

## Data Availability

The data contain personally identifiable information of the survey participants in the US and the UK and therefore cannot be made public. The anonymity of the survey participants is guaranteed and restricted by the informed consent and the IRB. For further requests, please contact the corresponding author.
