# Supplementary material for: Comparison of embryologist stress, somatization, and burnout reported by embryologists working in UK HFEA-licensed ART/IVF clinics and USA ART/IVF clinics
Source: Hum Reprod. 2024 Aug 28;39(10):2297–304. doi: 10.1093/humrep/deae191 (PMC11447060; doi:10.1093/humrep/deae191)
Supplement: deae191_Supplementary_Data_File_S1 [file deae191_supplementary_data_file_s1.pdf]

OXFORD
human reproduction
Human Reproduction, 2024, 1–1
https://doi.org/10.1093/humrep/deae191
Supplementary Data

Supplementary materials

Correlations between PSS and MBI Dimensions

There was a strong positive correlation between PSS and EX, a weaker positive correlation between PSS and CY, and a negative correlation between PSS and PE. The PHQ-15 scale was negatively correlated with all three MBI dimensions (Supplementary Figs S11 and S13).

Access to technology and automation

Multiple questions about double-work, cryostorage-related stress and anxiety (handling tanks and specimens), and if technology would lessen stress and ease the burden on the lab staff were asked.

1. **Technology use:** (1) Only 4 (1.6%) of 246 US Embryologists had access to an automated cryostorage platform; while the rest only had manually operated tanks and canes and goblets. One hundred fifty-two (62%) US embryologists used analog records and/or Excel spreadsheets, 49 (20%) reported having digital scanners with printed labels and Excel spreadsheets, and the rest (18%) used a combination of analog, EMR, and FileMaker records. (2) None of the 127 UK embryologists had access to an automated cryostorage platform; all had manually operated tanks and canes and goblets. Fifty-nine (46%) UK embryologists used analog records and/or Excel spreadsheets, 28 (22%) reported having digital scanners with printed labels and Excel spreadsheets, and the rest (32%) used a combination of analog, EMR, and FileMaker records.
2. **Double-Work Questions:** 72% of the US embryologists reported finding themselves doing double-work due to lack of technology integrations very often and often, and there were statistically significant differences in perceived stress between those who reported doing it very often vs all other three groups, e.g. often, rarely, and never (Supplementary Table S5A). Meanwhile, 58% of the UK embryologists reported doing it for the same reason, and there were no statistically significant differences in perceived stress between different groups (Supplementary Table S5B).
3. **Technology and Stress Questions:** 25% of the US embryologists responded that utilizing technology could completely lessen the stress they felt as an embryologist, and 20% of them responded that technology would completely ease the burden on the lab staff. Meanwhile, 15% of the UK Embryologists responded that utilizing technology could completely lessen the stress they felt as an embryologist, and 9% of them responded that technology would completely ease the burden on the lab staff (data not shown).
4. While not a scientific result word clouds in both populations named technology integration and less paperwork among the top items on their Wishlist. But technology themes were more prominent in the US study than in the UK study (data not shown).
