# Supplementary material for: Comparison of embryologist stress, somatization, and burnout reported by embryologists working in UK HFEA-licensed ART/IVF clinics and USA ART/IVF clinics
Source: Hum Reprod. 2024 Aug 28;39(10):2297–304. doi: 10.1093/humrep/deae191 (PMC11447060; doi:10.1093/humrep/deae191)
Supplement: deae191_Supplementary_Figure_S1 [file deae191_supplementary_figure_s1.pdf]

Human Reproduction, 2024, 1–1
https://doi.org/10.1093/humrep/deae191
Supplementary Data

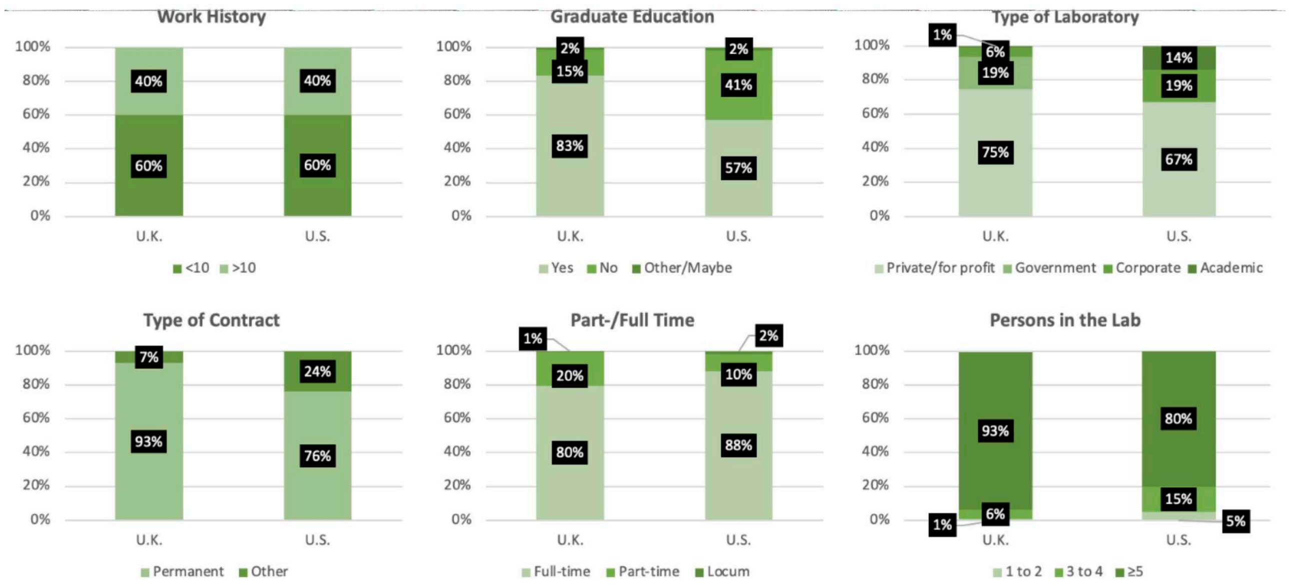

Supplementary Figure S1. **Employment characteristics of embryologists in UK and US ART/IVF clinics.**

- There is a similar breakdown of work experience by years in both countries: 60% with ≥10 years and 40% with ≤10 years of experience on the job.
- 83% of UK embryologists have a graduate degree because of the requirement they must have at least master's degree vs 57% of US embryologists.
- 75% of UK embryologists work for private/for profit clinics vs 67% in the US survey.
- 93% of UK embryologists work on a permanent basis vs 76% in the US survey.
- 80% of UK embryologists work full time vs 88% in the US survey.
- 93% of UK embryologists reported working in the labs with ≥5 people vs 80% in the US survey.
