# Supplementary material for: Comparison of embryologist stress, somatization, and burnout reported by embryologists working in UK HFEA-licensed ART/IVF clinics and USA ART/IVF clinics
Source: Hum Reprod. 2024 Aug 28;39(10):2297–304. doi: 10.1093/humrep/deae191 (PMC11447060; doi:10.1093/humrep/deae191)
Supplement: deae191_Supplementary_Figure_S2 [file deae191_supplementary_figure_s2.pdf]

OXFORD

human reproduction

Human Reproduction, 2024, 1–1
https://doi.org/10.1093/humrep/deae191
Supplementary Data

Employment Conditions	People		PSS		PHQ-15	
	#	%	Score	STD	Score	STD
**Work History, years[a]**						
>10	76	60%	17.74	6.50	8.55	4.95
≤10	51	40%	18.37	7.02	8.63	5.46
**Grand Total**	**127**	**100%**	**17.99**	**6.69**	**8.58**	**5.14**
**Graduate Degree[b]**						
Yes	105	83%	17.84	6.87	8.56	5.20
No	19	15%	18.42	5.81	8.58	5.32
Other/Maybe	2	2%	21.00	9.90	8.50	2.12
**Grand Total**	**126**	**100%**	**19.1**	**7.5**	**8.5**	**4.2**
**Type of Laboratory[c]**						
Private/for profit	95	75%	18.54	6.57	8.91	5.22
Government	24	19%	17.88	6.57	8.63	4.77
Corporate	7	6%	10.71	5.71	4.71	4.15
Academic	1	1%	20.00	NA	4.00	NA
**Grand Total**	**127**	**100%**	**17.99**	**6.69**	**8.58**	**5.14**
**Type of Contract[d]**						
Permanent	118	93%	18.33	6.77	8.72	5.20
Other	9	7%	13.56	3.54	6.78	3.99
**Grand Total**	**127**	**100%**	**17.99**	**6.69**	**8.58**	**5.14**
**Part-/Full-Time Employment[e]**						
Full-time	101	80%	18.14	6.74	8.68	5.25
Part-time	25	20%	17.48	6.75	8.40	4.73
Per diem	1	1%	16.00	NA	3.00	NA
**Grand Total**	**127**	**100%**	**17.99**	**6.69**	**8.58**	**5.14**
**Persons working in the laboratory[f]**						
1 to 2	1	1%	18.00	NA	9.00	NA
3 to 4	7	6%	24.29	5.77	14.00	8.08
≥5	117	93%	17.74	6.59	8.26	4.82
NA	**2**	**2%**	**11.00**	**5.66**	**8.50**	**4.95**
**Grand Total**	**126**	**100%**	**17.99**	**6.69**	**8.58**	**5.14**

Supplementary Figure S2. Employment conditions in the UK, PSS, and PHQ-15.

PSS and PHQ-15 with a statistically significant difference: *P*<0.05.

[a, b, e]None; [c] **PHQ-15**: Government vs Corporate; [d]**PSS**: Permanent vs Other; [f]**PSS**: 3–4 vs ≥5 people working in the laboratory.

Color coding: PSS: red—high, yellow—moderate, and light-green—low; PHQ-15: burgundy—high, deep-yellow—medium, green—low, and deep-green—minimal.
