# Supplementary material for: Comparison of embryologist stress, somatization, and burnout reported by embryologists working in UK HFEA-licensed ART/IVF clinics and USA ART/IVF clinics
Source: Hum Reprod. 2024 Aug 28;39(10):2297–304. doi: 10.1093/humrep/deae191 (PMC11447060; doi:10.1093/humrep/deae191)
Supplement: deae191_Supplementary_Figure_S3 [file deae191_supplementary_figure_s3.pdf]

OXFORD
human reproduction

Human Reproduction, 2024, 1–1
https://doi.org/10.1093/humrep/deae191
Supplementary Data

Employment Conditions	People		PSS		PHQ-15	
	#	%	Score	STD	Score	STD
**Work History, years[a]**						
>10	147	60%	16.97	5.26	9.56	4.84
≤10	97	39%	17.90	6.09	9.32	5.51
N/A	2	1%	21.50	0.71	21.00	4.24
**Grand Total**	**246**	**100%**	**18.79**	**4.02**	**13.29**	**4.86**
**Graduate Degree[b]**						
Yes	141	57%	16.86	5.25	10.11	4.94
No	101	41%	17.95	6.06	8.75	5.54
Maybe	4	2%	20.75	3.86	10.50	2.52
**Grand Total**	**246**	**100%**	**17.95**	**6.06**	**8.75**	**5.54**
**Type of Laboratory[c]**						
Academic	34	14%	17.41	3.94	8.06	8.06
Corporate	46	19%	17.20	4.98	10.93	10.93
Government	1	0%	20.00	0.00	14.00	14.00
Private/for profit	162	66%	17.46	6.10	9.57	9.57
N/A	3	1%	14.00	3.61	3.33	3.33
**Grand Total**	**246**	**100%**	**17.21**	**3.73**	**9.18**	**9.18**
**Type of Contract[d]**						
Permanent	187	76%	17.02	5.92	9.10	5.06
Other	56	23%	18.70	4.31	11.20	5.48
N/A	3	1%	14.67	2.08	7.67	2.31
**Grand Total**	**246**	**100%**	**16.79**	**4.10**	**9.32**	**4.28**
**Part-/Full-Time Employment[e]**						
Full-time	217	88%	17.53	5.75	9.49	5.10
Part-time	25	10%	16.80	3.97	9.60	5.82
Per diem	4	2%	12.50	4.80	13.25	6.65
**Grand Total**	**246**	**100%**	**15.61**	**4.84**	**10.78**	**5.86**
**Persons working in the laboratory[f]**						
1 to 2	12	5%	20.75	5.88	10.83	3.53
3 to 4	36	15%	18.67	6.07	8.39	5.53
≥5	198	80%	16.93	5.42	9.70	5.17
**Grand Total**	**246**	**100%**	**19.71**	**5.97**	**9.61**	**4.53**

Supplementary Figure S3. Employment conditions in the US, PSS, and PHQ-15.

PSS and PHQ-15 of working conditions with a statistically significant difference: $P<0.05$.

[a]**PHQ-15**: >10 vs N/A; and ≤10 vs N/A.

[b]**PHQ-15**: Yes vs No.

[c]**PHQ-15**: Academic vs Corporate; Academic vs N/A; and Corporate vs N/A.

[d]**PSS**: Permanent vs Other. **PHQ-15**: Permanent vs Other.

[e]No statistically significant differences.

[f]**PSS**: 1–2 vs ≥5 people working in the laboratory. **PHQ-15**: N/A.

Color coding: PSS: red—high, yellow—moderate, and light-green—low; PHQ-15: burgundy—high, deep-yellow—medium, green—low, and deep-green—minimal.
