# Supplementary material for: Comparison of embryologist stress, somatization, and burnout reported by embryologists working in UK HFEA-licensed ART/IVF clinics and USA ART/IVF clinics
Source: Hum Reprod. 2024 Aug 28;39(10):2297–304. doi: 10.1093/humrep/deae191 (PMC11447060; doi:10.1093/humrep/deae191)
Supplement: deae191_Supplementary_Figure_S5 [file deae191_supplementary_figure_s5.pdf]

OXFORD

human reproduction

Human Reproduction, 2024, 1–1
https://doi.org/10.1093/humrep/deae191
Supplementary Data

PSS Questions	People		PSS		PHQ-15	
	#	%	Score	STD	Score	STD
**In the last month, how often have you been upset because of something that happened unexpectedly?[a]**						
very often	6	5%	28.00	2.28	10.83	6.55
fairly often	12	9%	25.75	3.52	13.25	4.90
sometimes	56	44%	20.13	5.04	9.77	4.73
almost never	37	29%	13.89	3.63	6.86	3.92
never	16	13%	10.44	5.76	4.06	4.17
**Grand Total**	**127**	**100%**	**19.64**	**4.05**	**8.96**	**4.85**
**In the last month, how often have you felt that you were unable to control the important things in your life?[b]**						
very often	4	3%	27.75	3.30	14.00	5.60
fairly often	26	20%	24.27	5.16	10.69	4.66
sometimes	55	43%	18.95	4.31	9.40	5.14
almost never	31	24%	13.16	4.51	6.48	3.63
never	11	9%	8.45	3.96	3.45	4.16
**Grand Total**	**127**	**100%**	**18.52**	**4.25**	**8.81**	**4.64**
**In the last month, how often have you felt nervous and “stressed”?[c]**						
very often	26	20%	25.69	4.12	12.23	4.88
fairly often	39	31%	19.82	4.66	9.62	4.69
sometimes	40	31%	15.23	4.12	7.75	4.37
almost never	17	13%	11.76	4.83	4.82	3.57
never	5	4%	7.00	5.48	1.00	1.22
**Grand Total**	**127**	**100%**	**15.90**	**4.64**	**7.08**	**3.75**
**In the last month, how often have you felt confident about your ability to handle your personal problems?[d]**						
very often	22	17%	11.95	5.13	6.64	4.94
fairly often	57	45%	16.49	5.45	7.98	4.30
sometimes	39	31%	23.26	4.48	10.72	5.23
almost never	5	4%	25.00	6.96	11.40	8.38
never	4	3%	12.50	5.74	3.50	3.32
**Grand Total**	**127**	**100%**	**17.84**	**5.55**	**8.05**	**5.23**
**In the last month, how often have you felt that things were going your way?[e]**						
very often	9	7%	8.78	4.27	5.78	5.40
fairly often	47	37%	14.02	4.69	7.21	4.04
sometimes	52	41%	21.12	5.21	9.87	5.32
almost never	18	14%	24.06	5.01	10.17	5.83
never	1	1%	16.00	NA	3.00	NA
**Grand Total**	**9**	**7%**	**8.78**	**4.27**	**5.78**	**5.40**

PSS Questions	People		PSS		PHQ-15	
	#	%	Score	STD	Score	STD
**In the last month, how often have you found that you could not cope with all the things that you had to do?[f]**						
very often	11	9%	27.27	3.07	12.82	4.92
fairly often	25	20%	23.68	4.32	11.32	5.66
sometimes	52	41%	18.17	4.57	8.92	4.36
almost never	30	24%	12.53	3.13	6.20	3.39
never	9	7%	8.00	5.07	1.78	2.05
**Grand Total**	**127**	**100%**	**17.93**	**4.03**	**8.21**	**4.08**
**In the last month, how often have you felt that you were on top of things?[g]**						
very often	7	6%	11.43	5.77	5.71	3.95
fairly often	44	35%	13.30	5.09	6.36	4.03
sometimes	51	40%	20.25	5.28	10.18	5.20
almost never	22	17%	23.45	4.98	10.55	5.40
never	3	2%	23.67	8.02	6.33	5.77
**Grand Total**	**127**	**127**	**18.42**	**5.83**	**7.83**	**4.87**
**In the last month, how often have you been able to control irritations in your life?[h]**						
very often	11	9%	9.64	5.24	4.91	4.61
fairly often	48	38%	15.31	5.24	7.52	4.26
sometimes	54	43%	20.78	5.40	9.67	5.01
almost never	12	9%	25.00	4.94	12.50	5.87
never	2	2%	11.00	7.07	1.50	2.12
**Grand Total**	**127**	**100%**	**16.35**	**5.58**	**7.22**	**4.37**
**In the last month, how often have you been angered because of things that were outside your control?[i]**						
very often	10	8%	25.80	5.79	12.00	5.89
fairly often	28	22%	24.14	3.55	12.21	4.89
sometimes	51	40%	18.02	4.35	8.22	4.07
almost never	27	21%	12.78	3.67	6.44	4.14
never	11	9%	7.91	4.35	3.18	4.00
**Grand Total**	**127**	**100%**	**17.73**	**4.34**	**8.41**	**4.60**
**In the last month, how often have you felt difficulties were piling up so high that you could not overcome them?[j]**						
very often	8	6%	28.75	3.01	14.25	5.23
fairly often	20	16%	23.40	4.06	10.05	4.47
sometimes	47	37%	20.15	4.41	9.17	5.19
almost never	32	25%	14.19	3.35	8.03	4.04
never	20	16%	9.30	4.12	4.35	4.15
**Grand Total**	**127**	**100%**	**19.16**	**3.79**	**9.17**	**4.62**

Supplementary Figure S5. Perceived stress symptoms among embryologists in UK ART/IVF clinics, PSS and PHQ-15.

PSS and PHQ-15 within each question with a statistically significant difference: $P<0.05$.

[a]**PSS**: All are statistically different except for Very Often vs Fairly Often.

[b]**PSS**: All are statistically different.

[c]**PSS**: All are statistically different except for Almost Never vs Never.

[d]**PSS**: All are statistically different except for Very Often vs Never, Fairly Often vs Almost Never, Fairly Often vs Never, and Sometimes vs Almost Never.

[e]**PSS**: All are statistically different.

[f]**PSS**: All are statistically different.

[g]**PSS**: All are statistically different except for Very Often vs Fairly Often, Very Often vs Never, Fairly Often vs Never, Sometimes vs Never, and Almost Never vs Never.

[h]**PSS**: All are statistically different except for Very Often vs Never, Fairly Often vs Never, Sometimes vs Never, and Almost Never vs Never.

[i]**PSS**: All are statistically different.

[j]**PSS**: All are statistically different

Color coding: PSS: Red—high, yellow—moderate, and light-green—low; PHQ-15: burgundy—high, deep-yellow—medium, green—low, and deep-green—minimal.
