# Supplementary material for: Comparison of embryologist stress, somatization, and burnout reported by embryologists working in UK HFEA-licensed ART/IVF clinics and USA ART/IVF clinics
Source: Hum Reprod. 2024 Aug 28;39(10):2297–304. doi: 10.1093/humrep/deae191 (PMC11447060; doi:10.1093/humrep/deae191)
Supplement: deae191_Supplementary_Figure_S6 [file deae191_supplementary_figure_s6.pdf]

OXFORD

human reproduction

Human Reproduction, 2024, 1–2
https://doi.org/10.1093/humrep/deae191
Supplementary Data

PSS Questions	People #	People %	PSS Score	PSS STD	PHQ-15 Score	PHQ-15 STD
**In the last month, how often have you been upset because of something that happened unexpectedly?[a]**						
very often	11	4%	22.64	6.53	13.27	6.10
fairly often	54	22%	22.15	4.98	11.89	5.28
sometimes	112	46%	17.32	3.71	8.71	4.70
almost never	58	24%	13.40	4.52	8.67	5.01
never	11	4%	10.09	4.64	7.82	5.33
**Grand Total**	**246**	**100%**	**17.12**	**4.88**	**10.07**	**5.28**
**In the last month, how often have you felt that you were unable to control the important things in your life?[b]**						
very often	6	2%	27.17	8.66	11.83	4.92
fairly often	38	15%	22.26	5.12	13.24	5.52
sometimes	97	39%	19.20	3.60	10.06	4.65
almost never	91	37%	13.93	4.50	7.55	5.63
never	14	6%	9.57	3.49	8.21	4.61
**Grand Total**	**246**	**100%**	**18.43**	**5.07**	**10.18**	**5.07**
**In the last month, how often have you felt nervous and “stressed”?[c]**						
very often	31	13%	23.94	5.10	12.19	3.90
fairly often	75	30%	19.43	4.65	10.55	5.19
sometimes	102	41%	15.63	4.10	8.54	4.98
almost never	33	13%	13.21	4.27	8.36	5.64
never	5	2%	8.80	5.63	7.20	6.61
**Grand Total**	**246**	**100%**	**16.20**	**4.75**	**9.37**	**5.26**
**In the last month, how often have you felt confident about your ability to handle your personal problems?[d]**						
very often	51	21%	12.31	5.66	7.45	4.99
fairly often	97	39%	16.37	4.07	8.77	5.09
sometimes	79	32%	20.95	3.63	11.41	4.21
almost never	17	7%	22.12	6.19	12.65	6.52
never	2	1%	13.00	4.24	2.50	0.71
**Grand Total**	**246**	**100%**	**16.95**	**4.76**	**8.56**	**4.30**
**In the last month, how often have you felt that things were going your way?[e]**						
very often	19	8%	9.11	3.30	8.37	4.57
fairly often	90	37%	14.69	3.84	8.31	4.85
sometimes	117	48%	19.50	4.24	10.45	5.20
almost never	19	8%	25.26	4.90	11.53	5.95
never	1	0%	16.00	0.00	3.00	0.00
**Grand Total**	**246**	**100%**	**16.91**	**3.26**	**8.33**	**4.11**

PSS Questions	People #	People %	PSS Score	PSS STD	PHQ-15 Score	PHQ-15 STD
**In the last month, how often have you found that you could not cope with all the things that you had to do?[f]**						
very often	9	4%	28.44	5.15	14.33	3.32
fairly often	37	15%	22.38	3.09	12.51	5.28
sometimes	99	40%	18.65	3.87	10.05	4.94
almost never	79	32%	13.96	3.66	7.70	5.13
never	22	9%	10.91	5.30	7.14	4.94
**Grand Total**	**246**	**100%**	**18.87**	**4.21**	**10.35**	**4.72**
**In the last month, how often have you felt that you were on top of things?[g]**						
very often	26	11%	12.35	5.04	6.92	5.31
fairly often	86	35%	14.73	4.40	8.47	4.68
sometimes	109	44%	19.16	4.42	10.68	5.00
almost never	23	9%	23.83	4.71	11.48	5.98
never	2	1%	24.50	12.02	8.00	7.07
**Grand Total**	**246**	**100%**	**18.91**	**6.12**	**9.11**	**5.61**
**In the last month, how often have you been able to control irritations in your life?[h]**						
very often	20	8%	11.10	5.97	6.70	4.91
fairly often	92	37%	14.83	4.48	8.65	5.21
sometimes	107	43%	19.53	3.95	10.48	4.80
almost never	23	9%	22.39	5.80	11.13	5.30
never	3	1%	22.00	11.27	9.67	9.87
NA	1	0%	16.00	NA	16.00	NA
**Grand Total**	**246**	**100%**	**17.64**	**17.64**	**10.44**	**6.02**
**In the last month, how often have you been angered because of things that were outside your control?[i]**						
very often	9	4%	25.00	6.60	13.78	7.31
fairly often	52	21%	21.60	4.21	10.96	4.39
sometimes	111	45%	17.50	4.18	9.45	4.99
almost never	64	26%	13.89	4.80	8.69	5.47
never	10	4%	9.30	4.47	5.30	3.16
**Grand Total**	**246**	**100%**	**17.46**	**4.85**	**9.64**	**5.06**
**In the last month, how often have you felt difficulties were piling up so high that you could not overcome them?[j]**						
very often	6	2%	30.83	3.37	16.83	3.43
fairly often	35	14%	23.20	3.49	12.54	3.89
sometimes	98	40%	18.59	3.70	10.89	5.02
almost never	82	33%	14.61	3.92	7.17	4.49
never	21	9%	9.67	3.55	5.67	3.28
NA	4	2%	13.25	3.10	9.50	NA

Supplementary Figure S6. Perceived stress symptoms among embryologists in US ART/IVF clinics, PSS and PHQ-15.

PSS and PHQ-15 within each question with a statistically significant difference: $P<0.05$.

[a]**PSS**: Very Often vs Sometimes Very Often vs Almost Never, Very Often vs Never, Fairly Often vs Sometimes, Fairly Often vs Almost Never; Fairly Often vs Never, Sometimes vs Almost Never; Sometimes vs Never, Almost Never vs Never. **PHQ-15**: Very often vs Sometimes, Very Often vs Almost Never; Very Often vs Never, Fairly Often vs Sometimes, Fairly Often vs Almost Never; Fairly Often vs Never.

[b]**PSS**: Very Often vs Almost Never, Very Often vs Never, Fairly Often vs Sometimes, Fairly Often vs Almost Never; Fairly Often vs Never, Sometimes vs Almost Never; Sometimes vs Never, Almost Never vs Never. PHQ-15: Fairly Often vs Sometimes, Fairly Often vs Almost Never; Fairly Often vs Never, Sometimes vs Almost Never.

[c]**PSS**: Very Often vs Fairly Often, Very Often vs Sometimes, Very Often vs Almost Never; Very Often vs Never, Fairly Often vs Sometimes, Fairly Often vs Almost Never; Fairly Often vs Never, Sometimes vs Almost Never. **PHQ-15**: Very Often vs Sometimes, Very Often vs Almost Never; Fairly Often vs Sometimes.

[d]**PSS**: Very often vs Fairly Often, Very Often vs Sometimes, Very Often vs Almost Never; Fairly Often vs Sometimes, Fairly Often vs Almost Never. **PHQ-15**: Very Often vs Sometimes, Very Often vs Almost Never; Very Often vs Never, Fairly Often vs Sometimes, Fairly Often vs Almost Never, Fairly Often vs Never, Fairly Often vs Never.

(continued)

Supplementary Figure S6. **Continued**

[e]**PSS**: Very Often vs Fairly Often, Very Often vs Sometimes, Very Often vs Almost Never, Fairly Often vs Sometimes, Fairly Often vs Almost Never, Sometimes vs Almost Never. **PHQ-15**: Fairly Often vs Sometimes and Fairly Often vs Almost Never.

[f]**PSS**: Very often vs Fairly Often, Very Often vs Sometimes, Very Often vs Almost Never, Very Often vs Never, Fairly Often vs Sometimes, Fairly Often vs Almost Never, Fairly Often vs Never, Sometimes vs Almost Never, Sometimes vs Never, Almost Never vs Never. **PHQ-15**: Very Often vs Sometimes, Very Often vs Almost Never; Very Often vs Never, Fairly Often vs Sometimes, Fairly Often vs Almost Never, Sometimes vs Almost Never, Almost Never vs Never.

[g]**PSS**: Very Often vs Fairly Often, Very Often vs Sometimes, Very Often vs Almost Never, Fairly Often vs Sometimes, Fairly Often vs Almost Never, Sometimes vs Almost Never. **PHQ-15**: Very Often vs Sometimes, Very Often vs Almost Never, Fairly Often vs Sometimes, Fairly Often vs Almost Never.

[h]**PSS**: Very Often vs Fairly Often, Very Often vs Sometimes, Very Often vs Almost Never, Fairly Often vs Sometimes, Fairly Often vs Almost Never, Sometimes vs Almost Never. **PHQ-15**: Very Often vs Sometimes, Very Often vs Almost Never, Fairly Often vs Sometimes, Fairly Often vs Almost Never.

[i]**PSS**: Very Often vs Sometimes, Very Often vs Almost Never; Very Often vs Never, Fairly Often vs Sometimes, Fairly Often vs Almost Never; Fairly Often vs Never, Sometimes vs Almost Never, Sometimes vs Never, Almost Never vs Never. **PHQ-15**: Very Often vs Never, Fairly Often vs Sometimes, Fairly Often vs Almost Never; Fairly Often vs Never, Sometimes vs Never, Almost Never vs Never.

[j]**PSS**: Very Often vs Often; Very Often vs Sometimes, Very Often vs Almost Never, Very Often vs Never, Very Often vs N/A, Fairly Often vs Sometimes, Fairly Often vs Almost Never; Fairly Often vs Never, Sometimes vs Almost Never, Sometimes vs Never, Sometimes vs N/A, Almost Never vs Never. **PHQ-15**: Very Often vs Fairly Often, Very Often vs Sometimes, Very Often vs Almost Never, Very Often vs Never, Fairly Often vs Sometimes, Fairly Often vs Almost Never; Fairly Often vs Never, Sometimes vs Almost Never; Sometimes vs Never.

Color coding: PSS: Red—high, yellow—moderate, and light-green—low; PHQ-15: burgundy—high, deep-yellow—medium, green—low, and deep-green—minimal.
