# Supplementary material for: Comparison of embryologist stress, somatization, and burnout reported by embryologists working in UK HFEA-licensed ART/IVF clinics and USA ART/IVF clinics
Source: Hum Reprod. 2024 Aug 28;39(10):2297–304. doi: 10.1093/humrep/deae191 (PMC11447060; doi:10.1093/humrep/deae191)
Supplement: deae191_Supplementary_Figure_S8 [file deae191_supplementary_figure_s8.pdf]

OXFORD

human reproduction

Human Reproduction, 2024, 1–1

https://doi.org/10.1093/humrep/deae191

Supplementary Data

Somatic Symptoms	People		PSS		PHQ-15	
	#	%	Score	STD	Score	STD
**Stomach Pain[a]**						
bothered a lot	11	9%	23.27	6.17	15.55	4.27
bothered a little	41	32%	18.98	6.51	10.68	4.99
not bothered at all	75	59%	16.68	6.47	6.41	3.82
**Grand Total**	**127**	**100%**	**19.64**	**6.38**	**10.88**	**4.36**
**Back pain[b]**						
bothered a lot	34	27%	20.65	5.75	12.65	4.23
bothered a little	56	44%	18.02	5.50	8.79	4.39
not bothered at all	37	29%	15.51	8.21	4.54	3.75
**Grand Total**	**127**	**100%**	**18.06**	**6.49**	**8.66**	**4.12**
**Pain in your arms, legs, or joints (knees, hips, etc.)[c]**						
bothered a lot	19	15%	21.89	5.29	13.68	5.23
bothered a little	49	39%	17.98	6.44	9.63	4.67
not bothered at all	59	46%	16.75	6.91	6.07	3.87
**Grand Total**	**127**	**100%**	**18.87**	**6.21**	**9.79**	**4.59**
**Menstrual cramps or other problems with your period (women only)[d]**						
bothered a lot	23	18%	20.17	5.84	12.22	4.88
bothered a little	38	30%	18.71	7.01	10.29	4.34
not bothered at all	66	52%	16.82	6.63	6.33	4.57
**Grand Total**	**127**	**100%**	**18.57**	**6.50**	**9.61**	**4.60**
**Headaches[e]**						
bothered a lot	18	14%	23.17	6.49	14.50	5.40
bothered a little	65	51%	18.43	6.09	8.92	4.05
not bothered at all	44	35%	15.23	6.33	5.66	4.22
**Grand Total**	**127**	**100%**	**18.94**	**6.31**	**9.69**	**4.56**
**Chest pain[f]**						
bothered a lot	3	2%	29.67	2.08	18.00	4.36
bothered a little	23	18%	20.48	6.27	12.83	5.21
not bothered at all	101	80%	17.08	6.43	7.34	4.31
**Grand Total**	**127**	**100%**	**22.41**	**4.93**	**12.72**	**4.63**
**Dizziness[g]**						
bothered a lot	4	3%	25.50	4.65	17.50	3.87
bothered a little	35	28%	20.71	6.12	12.66	4.55
not bothered at all	88	69%	16.57	6.47	6.56	3.89
**Grand Total**	**127**	**100%**	**20.93**	**5.75**	**12.24**	**4.10**
**Fainting spells[h]**						
bothered a lot	0	0%	NA	NA		NA
bothered a little	11	9%	21.18	3.89	14.36	5.80
not bothered at all	116	91%	17.69	6.84	8.03	4.74
**Grand Total**	**127**	**100%**	**19.44**	**5.36**	**11.20**	**5.27**

Somatic Symptoms	People		PSS		PHQ-15	
	#	%	Score	STD	Score	STD
**Feeling your heart pound and race[i]**						
bothered a lot	8	6%	22.13	5.87	16.25	6.48
bothered a little	55	43%	20.44	5.92	10.67	4.27
not bothered at all	64	50%	15.38	6.44	5.83	3.68
**Grand Total**	**127**	**100%**	**19.31**	**6.07**	**10.92**	**4.81**
**Shortness of breath[j]**						
bothered a lot	2	2%	26.00	4.24	21.50	2.12
bothered a little	23	18%	20.70	6.26	13.09	4.45
not bothered at all	102	80%	17.23	6.61	7.31	4.36
**Grand Total**	**127**	**2%**	**21.31**	**5.70**	**13.97**	**3.64**
**Pain or problems during sexual intercourse[k]**						
bothered a lot	2	2%	25.50	3.54	16.50	9.19
bothered a little	21	17%	21.24	5.32	13.43	4.68
not bothered at all	104	82%	17.19	6.73	7.45	4.47
**Grand Total**	**127**	**100%**	**21.31**	**5.20**	**12.46**	**6.11**
**Constipation, loose bowels, or diarrhea[l]**						
bothered a lot	17	13%	22.53	5.79	14.29	4.61
bothered a little	39	31%	18.00	6.17	10.44	4.51
not bothered at all	71	56%	16.90	6.79	6.20	4.01
**Grand Total**	**127**	**100%**	**19.14**	**6.25**	**10.31**	**4.37**
**Nausea, gas, or indigestion[m]**						
bothered a lot	15	12%	20.33	6.56	15.40	3.79
bothered a little	51	40%	19.67	6.29	10.43	4.10
not bothered at all	61	48%	16.02	6.58	5.36	3.61
**Grand Total**	**127**	**100%**	**18.67**	**6.48**	**10.40**	**3.83**
**Feeling tired or low energy[n]**						
bothered a lot	52	41%	22.42	5.20	12.50	4.47
bothered a little	56	44%	16.18	5.35	7.00	3.17
not bothered at all	19	15%	11.21	5.85	2.53	2.57
**Grand Total**	**127**	**100%**	**16.60**	**5.47**	**7.34**	**3.40**
**Trouble sleeping[o]**						
bothered a lot	25	20%	23.36	5.41	13.84	4.66
bothered a little	61	48%	18.20	5.83	8.30	3.86
not bothered at all	41	32%	14.41	6.44	5.80	4.74
**Grand Total**	**127**	**100%**	**18.66**	**5.89**	**9.31**	**4.42**

Supplementary Figure S8. Perceived somatic symptom severity among embryologists in UK ART/IVF clinics, PSS and PHQ-15.

PSS within each somatic symptom with a statistically significant difference: $P < 0.05$.

[a,d,j,k] All are statistically significantly different except for Bothered a Lot vs Bothered a Little and Bothered a Little vs Not Bothered at All.

[b,c,l] All are statistically significantly different except for Bothered a Little vs Not Bothered at All.

[e,f,h,n,o] All statistically significantly different.

[g,i,m] All are statistically significantly different except for Bothered a Lot vs Bothered a Little.

Color coding: PSS: Red—high, yellow—moderate, and light-green—low; PHQ-15: burgundy—high, deep-yellow—medium, green—low, and deep-green—minimal.
