# Supplementary material for: Comparison of embryologist stress, somatization, and burnout reported by embryologists working in UK HFEA-licensed ART/IVF clinics and USA ART/IVF clinics
Source: Hum Reprod. 2024 Aug 28;39(10):2297–304. doi: 10.1093/humrep/deae191 (PMC11447060; doi:10.1093/humrep/deae191)
Supplement: deae191_Supplementary_Figure_S9 [file deae191_supplementary_figure_s9.pdf]

OXFORD

human reproduction

Human Reproduction, 2024, 1–2

https://doi.org/10.1093/humrep/deae191

Supplementary Data

Somatic Symptoms	People		PSS		PHQ-15	
	#	%	Score	STD	Score	STD
**Stomach Pain[a]**						
bothered a lot	17	7%	21.24	5.96	15.71	4.87
bothered a little	76	31%	19.42	5.43	12.64	4.20
not bothered at all	153	62%	15.92	5.14	7.35	4.29
**Grand Total**	**246**	**100%**	**17.67**	**5.28**	**10.00**	**5.20**
**Back pain[b]**						
bothered a lot	85	35%	18.92	5.37	13.20	4.45
bothered a little	105	43%	16.90	5.22	9.00	4.41
not bothered at all	56	22%	15.91	6.15	5.09	3.44
**Grand Total**	**246**	**100%**	**16.40**	**5.68**	**7.04**	**5.20**
**Pain in your arms, legs, or joints (knees, hips, etc.)[c]**						
bothered a lot	31	13%	20.03	6.19	14.71	5.01
bothered a little	134	54%	17.90	5.29	10.61	4.27
not bothered at all	81	33%	15.47	5.32	5.85	4.12
**Grand Total**	**246**	**100%**	**16.69**	5.30	**8.23**	4.19
**Menstrual cramps or other problems with your period (women only)[d]**						
bothered a lot	18	10%	20.33	6.14	14.50	3.87
bothered a little	62	34%	18.73	4.72	11.18	4.35
not bothered at all	103	56%	17.04	6.11	7.86	4.91
No Answer - Men	63	26%	15.73	4.81	9.33	5.46
**Grand Total**	**183**	**100%**	**17.16**	**5.60**	**9.56**	**5.20**
**Headaches[e]**						
bothered a lot	37	15%	22.08	5.46	13.76	4.52
bothered a little	122	50%	17.55	5.12	10.53	4.74
not bothered at all	87	35%	15.11	5.03	6.41	4.23
**Grand Total**	**246**	**100%**	**16.33**	**5.07**	**8.47**	**4.48**
**Chest pain[f]**						
bothered a lot	14	6%	19.21	4.58	18.50	4.26
bothered a little	67	27%	17.64	4.93	12.51	3.81
not bothered at all	165	67%	17.10	5.92	7.61	4.34
**Grand Total**	**246**	**100%**	**17.99**	**5.14**	**12.87**	**4.14**
**Dizziness[g]**						
bothered a lot	5	2%	22.00	8.46	20.60	5.18
bothered a little	75	30%	18.77	5.26	13.45	4.16
not bothered at all	166	67%	16.60	5.50	7.47	4.09
**Grand Total**	**246**	**100%**	**17.68**	**5.38**	**10.46**	**4.12**
**Fainting spells[h]**						
bothered a lot	12	5%	17.33	4.10	17.08	3.87
bothered a little	42	17%	17.12	3.88	12.88	3.31
not bothered at all	190	77%	17.43	6.03	8.38	4.91
N/A	2	1%	17.50	0.71	7.00	5.66
**Grand Total**	**246**	**100%**	**17.34**	**3.68**	**11.34**	**4.44**

Somatic Symptoms	People		PSS		PHQ-15	
	#	%	Score	STD	Score	STD
**Feeling your heart pound and race[i]**						
bothered a lot	25	10%	20.00	5.42	17.24	4.13
bothered a little	113	46%	17.93	5.23	10.92	3.64
not bothered at all	108	44%	16.18	5.77	6.36	4.25
**Grand Total**	**246**	**100%**	**18.04**	**5.47**	**11.51**	**4.01**
**Shortness of breath[j]**						
bothered a lot	12	5%	19.83	6.25	19.08	2.71
bothered a little	83	34%	17.88	5.50	12.31	4.01
not bothered at all	151	61%	16.89	5.56	7.29	4.26
**Grand Total**	**246**	**100%**	**18.20**	**5.77**	**12.90**	**3.66**
**Pain or problems during sexual intercourse[k]**						
bothered a lot	14	6%	19.00	6.29	18.07	3.63
bothered a little	53	22%	17.91	4.68	12.68	3.74
not bothered at all	177	72%	16.94	5.73	7.95	4.57
N/A	2	1%	18.00	4.00	10.00	1.00
**Grand Total**	**246**	**100%**	**17.12**	**5.15**	**8.90**	**4.05**
**Constipation, loose bowels, or diarrhea[l]**						
bothered a lot	28	11%	19.71	7.01	15.39	4.86
bothered a little	105	43%	18.54	5.63	11.40	4.06
not bothered at all	113	46%	15.70	4.67	6.41	4.04
**Grand Total**	**246**	**100%**	**17.12**	**5.15**	**8.90**	**4.05**
**Nausea, gas, or indigestion[m]**						
bothered a lot	27	11%	21.07	6.55	16.22	4.38
bothered a little	113	46%	18.13	5.38	10.96	4.17
not bothered at all	106	43%	15.61	4.95	6.38	3.99
**Grand Total**	**246**	**100%**	**16.87**	**5.16**	**8.67**	**4.08**
**Feeling tired or low energy[n]**						
bothered a lot	95	39%	20.35	5.39	12.20	4.93
bothered a little	130	53%	15.65	4.89	8.39	4.45
not bothered at all	21	9%	14.57	4.88	4.86	4.88
**Grand Total**	**246**	**100%**	**15.11**	**4.89**	**6.62**	**4.66**
**Trouble sleeping[o]**						
bothered a lot	56	23%	21.73	5.31	12.93	5.09
bothered a little	120	49%	17.31	4.64	10.52	4.28
not bothered at all	69	28%	13.94	4.99	5.14	3.68
N/A	1	0%	17.00	0.00	11.00	0.00
**Grand Total**	**246**	**100%**	**16.08**	**3.21**	**8.89**	**2.65**

Supplementary Figure S9. Perceived somatic symptom severity among embryologists in US ART/IVF clinics, PSS and PHQ-15.

PSS and PHQ-15 within each somatic symptom with a statistically significant difference: $P < 0.05$.

[a]**PSS**: Bothered a Lot vs Bothered a Little; Bothered a Lot vs Not Bothered at All; and Bothered a Little vs Not Bothered at All. **PHQ-15**: Bothered a Lot vs Not Bothered at All; and Bothered a Little vs Not Bothered at All.

(continued)

Supplementary Figure S9. Continued

[b]**PSS**: Bothered a Lot vs Bothered a Little; and Bothered a Lot vs Not Bothered at All. **PHQ-15**: Bothered a Lot vs Bothered a Little; Bothered a Lot vs Not Bothered at All; and Bothered a Little vs Not Bothered at All.

[c]**PSS**: Bothered a Lot vs Not Bothered at All; and Bothered a Little vs Not Bothered at All. **PHQ-15**: Bothered a Lot vs Bothered a Little; Bothered a Lot vs Not Bothered at All; and Bothered a Little vs Not Bothered at All.

[d]**PSS**: Bothered a Lot vs Not Bothered at All; and Bothered a Little vs Not Bothered at All. **PHQ-15**: Bothered a Lot vs Bothered a Little; Bothered a Lot vs Not Bothered at All; and Bothered a Little vs Not Bothered at All.

[e]**PSS**: Bothered a Lot vs Bothered a Little; Bothered a Lot vs Not Bothered at All; and Bothered a Little vs Not Bothered at All. **PHQ-15**: Bothered a Lot vs Bothered a Little; Bothered a Lot vs Not Bothered at All; and Bothered a Little vs Not Bothered at All.

[f]**PSS**: N/A. **PHQ-15**: Bothered a Lot vs Bothered a Little; Bothered a Lot vs Not Bothered at All; and Bothered a Little vs Not Bothered at All.

[g]**PSS**: Bothered a Little vs Not Bothered at All. **PHQ-15**: Bothered a Lot vs Bothered a Little; Bothered a Lot vs Not Bothered at All; and Bothered a Little vs Not Bothered at All.

[h]**PSS**: N/A. **PHQ-15**: Bothered a Lot vs Bothered a Little; Bothered A Lot vs Not Bothered at All; and Bothered a Little vs Not Bothered at All.

[i]**PSS**: Bothered a Lot vs Not Bothered at All; and Bothered a Little not Bothered at All. **PHQ-15**: Bothered a Lot vs Not Bothered at All; Bothered a Lot vs Bothered a Little; and Bothered a Little vs Not Bothered at All.

[j]**PSS**: N/A. **PHQ-15**: Bothered a Lot vs Bothered a Little; Bothered a Lot vs Not Bothered at All; and Bothered a Little vs Not Bothered at All.

[k]**PSS**: N/A. **PHQ-15**: Bothered a Lot vs Bothered a Little; Bothered a Lot vs Not Bothered at All; Bothered a Lot vs N/A; and Bothered a Little vs Not Bothered at All.

[l]**PSS**: Bothered a Lot vs Not Bothered at All; and Bothered a Little vs Not Bothered at All. **PHQ-15**: Bothered a Lot vs Bothered a Little; Bothered a Lot vs Not Bothered at All; and Bothered a Little vs Not Bothered at All.

[m]**PSS**: Bothered a Lot vs Bothered a Little; Bothered a Lot vs Not Bothered at All; and Bothered a Little vs Not Bothered at All. **PHQ-15**: Bothered a Lot vs Bothered a Little; Bothered a Lot vs Not Bothered at All; and Bothered a Little vs Not Bothered at All.

[n]**PSS**: Bothered a Lot vs Bothered a Little; and Bothered a Lot vs Not Bothered at All. **PHQ-15**: Bothered a Lot vs Not Bothered at All; Bothered a Lot vs Bothered a Little; and Bothered a Little vs Not Bothered at All.

[o]**PSS**: Bothered a Lot vs Bothered a Little; Bothered a Lot vs Not Bothered at All; and Bothered a Little vs Not Bothered at All. **PHQ-15**: Bothered a Lot vs Bothered a Little; Bothered a Lot vs Not Bothered at All; and Bothered a Little vs Not Bothered at All.

Color coding: PSS: Red—high, yellow—moderate, and light-green—low; PHQ-15: burgundy—high, deep-yellow—medium, green—low, and deep-green—minimal.
