# Supplementary material for: Comparison of embryologist stress, somatization, and burnout reported by embryologists working in UK HFEA-licensed ART/IVF clinics and USA ART/IVF clinics
Source: Hum Reprod. 2024 Aug 28;39(10):2297–304. doi: 10.1093/humrep/deae191 (PMC11447060; doi:10.1093/humrep/deae191)
Supplement: deae191_Supplementary_Figure_S10 [file deae191_supplementary_figure_s10.pdf]

Human Reproduction, 2024, 1–1
https://doi.org/10.1093/humrep/deae191
Supplementary Data

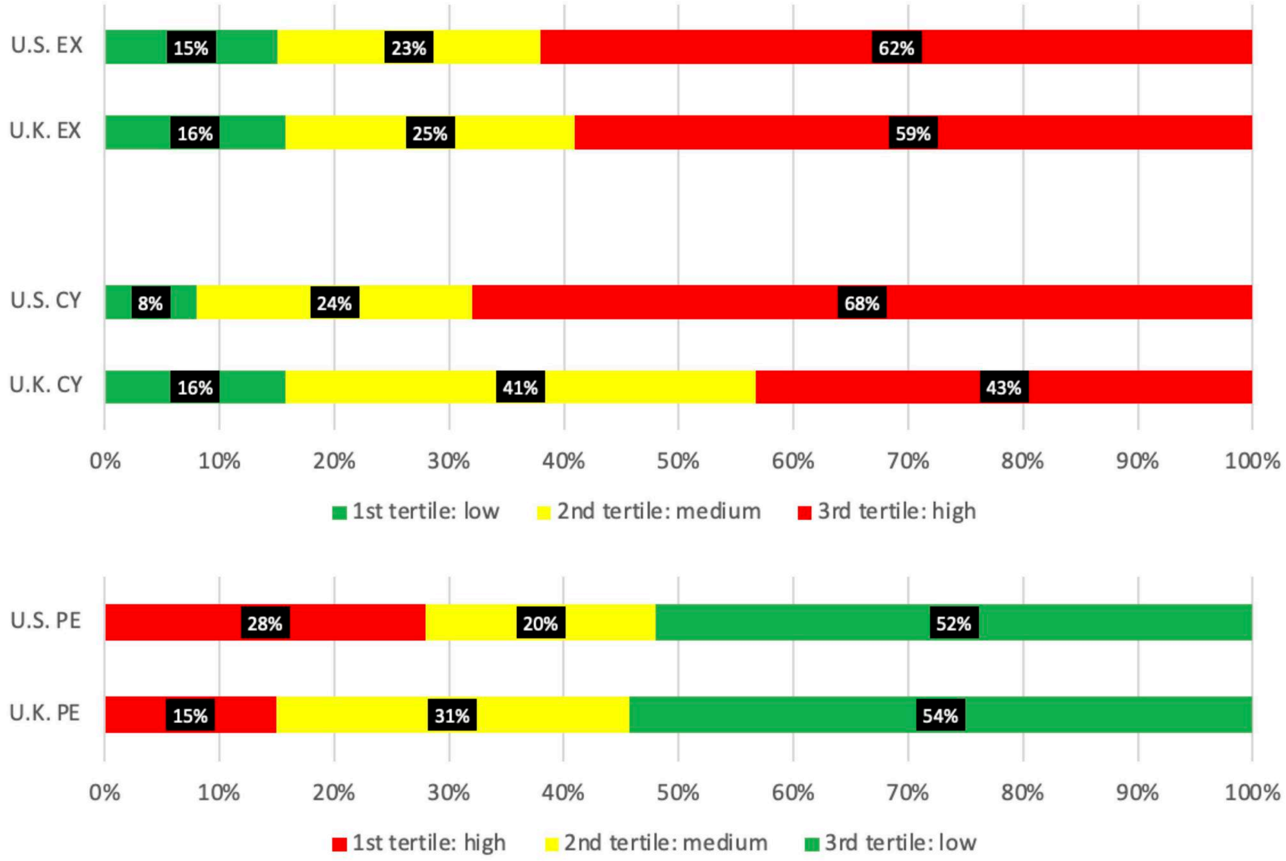

Supplementary Figure S10. Burnout levels among embryologists working in UK and US ART/IVF clinics measured on three MBI dimensions: EX, exhaustion; CY, cynicism; and PE, professional efficacy.
