# Supplementary material for: Comparison of embryologist stress, somatization, and burnout reported by embryologists working in UK HFEA-licensed ART/IVF clinics and USA ART/IVF clinics
Source: Hum Reprod. 2024 Aug 28;39(10):2297–304. doi: 10.1093/humrep/deae191 (PMC11447060; doi:10.1093/humrep/deae191)
Supplement: deae191_Supplementary_Figure_S11 [file deae191_supplementary_figure_s11.pdf]

OXFORD
human reproduction
Human Reproduction, 2024, 1–1
https://doi.org/10.1093/humrep/deae191
Supplementary Data

Burnout	People		PSS		PHQ-15	
	#	%	Score	STD	Score	STD
**MBI Exhaustion[a]**						
1st tertile ≤1.5	20	16%	11.90	4.83	5.00	4.24
2nd tertile 1.67–2.33	32	25%	16.25	5.61	7.69	4.50
3rd tertile ≥2.5	75	59%	20.36	6.33	9.92	5.12
**Grand Total**	**127**	**100%**	**16.17**	**5.59**	**7.54**	**4.62**
**MBI Cynicism[b]**						
1st tertile ≤0.5	20	16%	14.60	6.95	6.55	5.86
2nd tertile 0.75–1.5	52	41%	16.13	5.39	8.23	5.04
3rd tertile ≥1.75	55	43%	20.98	6.59	9.65	4.77
**Grand Total**	**127**	**100%**	**17.24**	**6.31**	**8.15**	**5.22**
**MBI Professional Efficacy[c]**						
1st tertile ≤4.33	19	15%	23.68	5.94	11.79	4.14
2nd tertile 4.5–5.17	39	31%	19.69	6.02	8.62	4.95
3rd tertile ≥5.33	69	54%	15.46	6.02	7.68	5.19
**Grand Total**	**127**	**100%**	**19.61**	**5.99**	**9.36**	**4.76**

Supplementary Figure S11. Burnout (MBI-GS tertiles), PSS and PHQ-15 among embryologists in UK ART/IVF clinics. In MBI EX and CY, first, second, and third tertiles correspond to low, medium, and high levels of burnout, respectively. In MBI PE, first, second, and third tertiles correspond to high, medium, and low, respectively. MBI values were calculated according to the MBI Manual, 3rd Edition, as per López-Lería *et al.* (2014).

PSS of burnout levels with a statistically significant difference: $P < 0.05$.

[a]**PSS**: 1st vs 2nd; 1st vs 3rd; and 2nd vs 3rd.

[b]**PSS**: 1st vs 3rd; 1st vs 2nd; and 2nd vs 3rd.

[c]**PSS**: 1st vs 3rd; 1st vs 2nd; and 2 vs 3rd

Color coding: PSS: red—high, yellow—moderate, and light-green—low. PHQ-15: burgundy—high, deep-yellow—medium, green—low, and deep-green—minimal.
