# Supplementary material for: Comparison of embryologist stress, somatization, and burnout reported by embryologists working in UK HFEA-licensed ART/IVF clinics and USA ART/IVF clinics
Source: Hum Reprod. 2024 Aug 28;39(10):2297–304. doi: 10.1093/humrep/deae191 (PMC11447060; doi:10.1093/humrep/deae191)
Supplement: deae191_Supplementary_Figure_S12 [file deae191_supplementary_figure_s12.pdf]

OXFORD

human reproduction

Human Reproduction, 2024, 1–1
https://doi.org/10.1093/humrep/deae191
Supplementary Data

Burnout	People		PSS		PHQ-15	
	#	%	Score	STD	Score	STD
**MBI Exhaustion[a]**						
1st tertile ≤1.5	36	15%	12.81	4.53	7.36	4.853
2nd tertile 1.67–2.33	57	23%	15.11	4.46	8.72	5.281
3rd tertile ≥2.5	153	62%	19.29	5.28	10.39	5.072
**Grand Total**	**246**	**100%**	**15.73**	**4.76**	**8.82**	**5.07**
**MBI Cynicism[b]**						
1st tertile ≤0.5	20	8%	12.85	5.66	5.90	4.12
2nd tertile 0.75–1.5	59	24%	14.80	4.72	7.31	4.60
3rd tertile ≥1.75	167	68%	18.82	5.28	10.80	5.06
**Grand Total**	**246**	**100%**	**15.49**	**5.60**	**9.56**	**5.20**
**MBI Professional Efficacy[c]**						
1st tertile ≤4.33	69	28%	18.54	5.05	11.61	5.11
2nd tertile 4.5–5.17	50	20%	18.04	4.87	9.82	5.10
3rd tertile ≥5.33	127	52%	16.47	6.02	8.35	4.95
**Grand Total**	**246**	**100%**	**17.68**	**5.31**	**9.93**	**5.05**

Supplementary Figure S12. Burnout (MBI-GS tertiles), PSS, and PHQ-15 among embryologists in US ART/IVF clinics. In MBI EX and CY, first, second, and third tertiles correspond to low, medium, and high levels of burnout, respectively. In MBI PE, first, second, and third tertiles correspond to high, medium, and low, respectively. MBI values were calculated according to the MBI Manual, 3rd Edition, as per López-Lería *et al.* (2014).

PSS and PHQ-15 of working conditions with a statistically significant difference: $P < 0.05$.

[a]**PSS**: first vs second; first vs third; and second vs third; **PHQ-15**: first vs third; and second vs third.

[b]**PSS**: 1st vs 3rd; and 2nd vs 3rd. **PHQ-15**: 1st vs 3rd; and 2nd vs 3rd.

[c]**PSS**: 1st vs 3rd. **PHQ-15**: 1st vs 3rd.

Color coding: PSS: Red—high, yellow—moderate, and light-green—low; PHQ-15: burgundy—high, deep-yellow—medium, green—low, and deep-green—minimal.
