# Supplementary material for: Comparison of embryologist stress, somatization, and burnout reported by embryologists working in UK HFEA-licensed ART/IVF clinics and USA ART/IVF clinics
Source: Hum Reprod. 2024 Aug 28;39(10):2297–304. doi: 10.1093/humrep/deae191 (PMC11447060; doi:10.1093/humrep/deae191)
Supplement: deae191_Supplementary_Figure_S13 [file deae191_supplementary_figure_s13.pdf]

OXFORD human reproduction

Human Reproduction, 2024, 1–2
https://doi.org/10.1093/humrep/deae191
Supplementary Data

Working Conditions	People		PSS		PHQ-15	
	#	%	Score	STD	Score	STD
**Working overtime[a]**						
Yes	103	81%	18.35	6.57	8.69	5.16
No	24	19%	16.46	7.13	8.13	5.11
**Grand Total**	**127**	**100%**	**17.41**	**6.85**	**8.41**	**5.14**
**Mandatory or voluntary overtime[b]**						
Mandatory	39	31%	18.33	6.24	9.00	6.00
Voluntary	88	69%	17.84	6.91	8.40	4.73
**Grand Total**	**127**	**100%**	**18.09**	**6.58**	**8.70**	**5.37**
**Compensated overtime[c]**						
Yes	86	68%	17.48	6.80	8.70	5.43
No	41	32%	19.07	6.42	8.34	4.53
**Grand Total**	**127**	**100%**	**18.28**	**6.61**	**8.52**	**4.98**
**Is compensation for working weekends and holidays appropriate?[d]**						
Yes	48	38%	16.10	6.48	7.25	4.80
No	61	48%	19.62	6.44	9.97	5.30
Maybe	13	10%	16.92	7.24	6.00	3.27
I don't know	5	4%	19.00	7.14	11.20	5.22
**Grand Total**	**127**	**100%**	**17.91**	**6.83**	**8.61**	**4.65**
**Taking two or more days off in a row during regular weeks[e]**						
Yes	79	62%	16.97	6.35	7.77	4.73
No	48	38%	19.67	6.97	9.92	5.55
**Grand Total**	**127**	**100%**	**18.32**	**6.66**	**8.85**	**5.14**
**Flexibility of scheduling in the laboratory[f]**						
Yes	65	51%	16.08	5.67	7.42	4.51
No	32	25%	19.09	8.47	10.28	5.48
Maybe	21	17%	21.71	4.96	9.24	5.97
I don't know	9	7%	19.22	6.00	9.44	4.77
**Grand Total**	**127**	**100%**	**19.03**	**6.28**	**9.10**	**5.18**
**Missing on key life events because of work[g]**						
Yes	45	35%	20.89	6.61	10.76	5.47
No	57	45%	15.91	6.03	7.21	4.72
Maybe	23	18%	17.35	6.91	7.48	4.14
I don't know	2	2%	19.50	0.71	11.50	4.95
**Grand Total**	**127**	**100%**	**18.41**	**5.07**	**9.24**	**4.82**
**Enough time to plan ahead for demands of the job[h]**						
Yes	64	50%	15.83	5.79	7.02	4.44
No	24	19%	23.38	6.02	11.21	5.43
Maybe	35	28%	18.11	6.92	9.29	5.34
I don't know	4	3%	19.25	4.92	11.75	4.35
**Grand Total**	**127**	**100%**	**19.14**	**5.91**	**9.82**	**4.89**

Supplementary Figure S13. Working conditions, life-work balance: PSS and PHQ-15 in the UK. PSS of working conditions with a statistically significant difference: $P < 0.05$.

(continued)

Supplementary Figure S13. **Continued**

[a,b,c]None.

[d]Yes vs No.

[e]Yes vs No.

[f]Yes vs No; Yes vs Maybe; and No vs I don’t know.

[g]Yes vs No; Yes vs Maybe; and No vs I Don’t Know.

[h]Yes vs No; and No vs Maybe.

Color coding: PSS: Red—high, yellow—moderate, and light-green—low; PHQ-15: burgundy—high, deep-yellow—medium, green—low, and deep-green—minimal.
