# Supplementary material for: Comparison of embryologist stress, somatization, and burnout reported by embryologists working in UK HFEA-licensed ART/IVF clinics and USA ART/IVF clinics
Source: Hum Reprod. 2024 Aug 28;39(10):2297–304. doi: 10.1093/humrep/deae191 (PMC11447060; doi:10.1093/humrep/deae191)
Supplement: deae191_Supplementary_Figure_S14 [file deae191_supplementary_figure_s14.pdf]

OXFORD
human reproduction
Human Reproduction, 2024, 1–1
https://doi.org/10.1093/humrep/deae191
Supplementary Data

**How often do you find yourself doing "double-work" in your lab due to lack of technology integrations and paper-based records?[i]**						
Very Often	28	22%	18.75	7.02	9.93	4.99
Often	46	36%	18.17	6.71	9.02	5.56
Rarely	47	37%	17.89	6.46	7.51	4.44
Never	6	5%	13.83	7.05	7.33	6.83
**Grand Total**	**127**	**100%**	**17.16**	**6.81**	**8.45**	**5.46**
**Does the number of on call embryologists meet the needs of your laboratory in case of emergencies?[j]**						
Yes	87	69%	17.03	6.67	8.01	5.08
No	15	12%	19.27	6.41	7.73	3.43
Maybe	13	10%	21.69	7.10	12.46	6.64
I don't know	12	9%	19.33	5.50	9.58	3.85
**Grand Total**	**127**	**100%**	**19.33**	**6.42**	**9.45**	**4.75**
**Does the possibility of being called in for an emergency cause you anxiety and the loss of sleep?[k]**						
Yes	58	46%	19.47	6.49	9.47	5.53
No	69	54%	16.75	6.66	7.84	4.70
**Grand Total**	**58**	**46%**	**19.47**	**6.49**	**9.47**	**5.53**
**Work unit grade (A–F)[l]**						
Excellent	49	39%	16.49	6.83	6.90	4.87
Very good	61	48%	18.56	6.36	9.03	4.60
Acceptable	13	10%	19.46	6.91	12.08	5.79
Failing	1	1%	32.00	NA	21.00	NA
Poor	3	2%	20.00	4.36	7.67	4.93
**Grand Total**	**127**	**100%**	**17.99**	**6.69**	**8.58**	**5.14**

Supplementary Figure S14. Working conditions, double-work, laboratory emergencies, and laboratory safety: PSS and PHQ-15 in the UK.

PSS of working conditions with a statistically significant difference: $P < 0.05$.

[a,b]None.

[c]Yes vs No

[d]Yes vs No.

Color coding: PSS: Red—high, yellow—moderate, and light-green—low; PHQ-15: burgundy—high, deep-yellow—medium, green—low, and deep-green—minimal.
