# Supplementary material for: Comparison of embryologist stress, somatization, and burnout reported by embryologists working in UK HFEA-licensed ART/IVF clinics and USA ART/IVF clinics
Source: Hum Reprod. 2024 Aug 28;39(10):2297–304. doi: 10.1093/humrep/deae191 (PMC11447060; doi:10.1093/humrep/deae191)
Supplement: deae191_Supplementary_Figure_S15 [file deae191_supplementary_figure_s15.pdf]

OXFORD

human reproduction

Human Reproduction, 2024, 1–2
https://doi.org/10.1093/humrep/deae191
Supplementary Data

Working Conditions	People		PSS		PHQ-15	
	#	%	Score	STD	Score	STD
**Working overtime[a]**						
Yes	196	80%	17.44	5.69	9.96	5.23
No	50	20%	17.10	5.28	7.98	4.68
**Grand Total**	**246**	**100%**	**17.10**	**5.28**	**7.98**	**4.68**
**Mandatory or voluntary overtime[b]**						
Mandatory	116	47%	18.01	5.70	9.11	5.06
Voluntary	127	52%	16.76	5.45	9.99	5.31
N/A	3	1%	18.33	7.57	8.67	6.35
**Grand Total**	**246**	**100%**	**17.70**	**6.24**	**9.26**	**5.57**
**Compensated overtime[c]**						
Yes	104	42%	16.79	5.63	10.49	4.85
No	140	57%	17.86	5.54	8.96	5.35
N/A	2	1%	13.00	7.07	3.50	0.71
**Grand Total**	**246**	**100%**	**15.43**	**6.30**	**6.23**	**3.03**
**Is compensation for working weekends and holidays appropriate?[d]**						
Yes	45	18%	13.87	4.60	6.18	4.11
No	124	50%	19.18	5.98	9.56	5.15
Maybe	59	24%	16.49	4.57	11.85	4.57
I don't know	18	7%	16.56	2.81	10.50	5.74
**Grand Total**	**246**	**100%**	**13.87**	**4.60**	**6.18**	**4.11**
**Taking two or more days off in a row during regular weeks[e]**						
Yes	95	39%	16.17	5.91	8.46	5.36
No	147	60%	18.12	5.28	10.29	4.95
N/A	4	2%	18.25	5.91	9.00	6.83
**Grand Total**	**246**	**100%**	**18.19**	**5.60**	**9.64**	**5.89**
**Flexibility of scheduling in the laboratory[f]**						
Yes	115	47%	15.94	5.59	8.63	5.23
No	52	21%	20.17	5.56	10.46	4.79
Maybe	70	28%	17.63	5.03	10.60	5.28
I don't know	9	4%	17.44	4.48	8.22	4.63
**Grand Total**	**246**	**100%**	**17.44**	**4.48**	**8.22**	**4.63**
**Missing on key life events because of work[g]**						
Yes	139	57%	18.58	5.57	10.09	5.33
No	50	20%	14.58	6.47	6.78	4.41
Maybe	49	20%	16.73	3.81	10.80	4.75
I don't know	8	3%	17.63	2.56	10.25	4.77
**Grand Total**	**246**	**100%**	**17.37**	**5.60**	**9.56**	**5.20**

Supplementary Figure S15. Working conditions, life-work balance: PSS and PHQ-15 in the US. PSS and PHQ-15 of working conditions with a statistically significant difference: $P < 0.05$.

[a]**PSS**: N/A. **PHQ-15**: Yes vs No.

[b]No statistically significant differences.

(continued)

Supplementary Figure S15. **Continued**

[c]**PSS**: N/A. **PHQ-15**: Yes vs N/A; Yes vs No; and No vs N/A.

[d]**PSS**: Yes vs No; Yes vs Maybe; Yes vs I Don't Know; No vs I Don't Know; and No vs Maybe. **PHQ-15**: Yes vs No; Yes vs Maybe; Yes vs I Don't Know; and No vs Maybe.

[e]**PSS**: Yes vs No. **PHQ-15**: Yes vs No.

[f]**PSS**: Yes vs No; Yes vs Maybe; and No vs Maybe. **PHQ-15**: Yes vs No; and Yes vs Maybe.

[g]**PSS**: Yes vs No; Yes vs Maybe; No vs I Don't Know; and No vs Maybe. **PHQ-15**: Yes vs No; and No vs Maybe.

Color coding: PSS: Red—high, yellow—moderate, and light-green—low; PHQ-15: burgundy—high, deep-yellow—medium, green—low, and deep-green—minimal.
