# Supplementary material for: Comparison of embryologist stress, somatization, and burnout reported by embryologists working in UK HFEA-licensed ART/IVF clinics and USA ART/IVF clinics
Source: Hum Reprod. 2024 Aug 28;39(10):2297–304. doi: 10.1093/humrep/deae191 (PMC11447060; doi:10.1093/humrep/deae191)
Supplement: deae191_Supplementary_Figure_S16 [file deae191_supplementary_figure_s16.pdf]

OXFORD

human reproduction

Human Reproduction, 2024, 1–1
https://doi.org/10.1093/humrep/deae191
Supplementary Data

**Enough time to plan ahead for demands of the job[h]**						
Yes	91	37%	15.97	5.55	8.00	4.80
No	49	20%	20.04	5.55	10.37	5.41
Maybe	94	38%	17.43	5.47	10.79	5.07
I don't know	12	5%	16.67	3.17	8.50	5.50
**Grand Total**	**246**	**100%**	**18.00**	**5.55**	**9.18**	**5.11**
**How often do you find yourself doing "double-work" in your lab due to lack of technology integrations and paper-based records?[i]**						
Very Often	70	28%	19.27	5.17	11.10	5.36
Often	106	43%	16.77	4.73	8.85	4.28
Rarely	63	26%	16.60	5.43	9.19	5.12
Never	6	2%	13.83	2.48	9.17	4.40
N/A	1	0%	17.00	N/A	3.00	N/A
**Grand Total**	**246**	**3%**	**15.42**	**2.48**	**6.08**	**4.40**
**Does the number of on call embryologists meet the needs of your laboratory in case of emergencies?[j]**						
Yes	120	49%	16.60	5.47	8.48	4.65
No	33	13%	18.45	4.53	11.30	5.62
I don't know	30	12%	18.60	5.84	9.07	4.97
Maybe	62	25%	17.73	4.05	10.97	4.38
N/A	1	0%	15.00	N/A	9.00	N/A
**Grand Total**	**246**	**100%**	**17.37**	**5.09**	**9.56**	**4.88**
**Does the possibility of being called in for an emergency cause you anxiety and the loss of sleep?[k]**						
Yes	92	37%	18.92	5.09	10.70	4.97
No	150	61%	16.46	4.99	8.93	4.71
N/A	4	2%	15.75	1.26	7.25	5.03
**Grand Total**	**246**	**100%**	**17.37**	**5.09**	**9.56**	**4.88**
**Work unit grade (A–F)[l]**						
Excellent	70	28%	15.76	5.61	7.86	4.79
Very good	107	43%	17.83	5.60	9.86	5.40
Acceptable	60	24%	17.65	4.69	10.82	4.90
Poor	6	2%	26.67	4.97	13.83	2.99
Failing	3	1%	14.33	2.08	5.00	1.00
**Grand Total**	**246**	**100%**	**19.55**	**3.91**	**9.88**	**2.97**

Supplementary Figure S16. Working conditions, double-work, laboratory emergencies, and laboratory safety: PSS and PHQ-15 in the US.

PSS and PHQ-15 of working conditions with a statistically significant difference: $P < 0.05$.

[a]**PSS**: Yes vs No; No vs I Don't Know; and No vs Maybe. **PHQ-15**: Yes vs No; and Yes vs Maybe.

[b,c,d] None.

Color coding: PSS: Red—high, yellow—moderate, and light-green—low; PHQ-15: burgundy—high, deep-yellow—medium, green—low, and deep-green—minimal.
