# Supplementary material for: Comparison of embryologist stress, somatization, and burnout reported by embryologists working in UK HFEA-licensed ART/IVF clinics and USA ART/IVF clinics
Source: Hum Reprod. 2024 Aug 28;39(10):2297–304. doi: 10.1093/humrep/deae191 (PMC11447060; doi:10.1093/humrep/deae191)
Supplement: deae191_Supplementary_Figure_S17 [file deae191_supplementary_figure_s17.pdf]

OXFORD

human reproduction

Human Reproduction, 2024, 1–1

https://doi.org/10.1093/humrep/deae191

Supplementary Data

Level of Anxiety	People		PSS		PHQ-15	
	#	%	Score	STD	Score	STD
Constant	3	2%	31.33	1.15	13.00	8.00
High	22	17%	22.95	4.63	10.45	5.32
Moderate	34	27%	19.71	5.85	9.91	4.67
Mild	50	39%	16.06	5.14	8.04	4.67
No	18	14%	11.83	6.53	4.56	4.30
**Grand Total**	**127**	**100%**	**20.38**	**4.66**	**9.19**	**5.39**

Supplementary Figure S17. Levels of anxiety re: Cryostorage, PSS, and PHQ-15 in the UK.

PSS and PHQ-15 of working conditions with a statistically significant difference: $P < 0.05$.

PSS: Constant vs High Anxiety; Constant vs Moderate Anxiety; Constant vs Mild Anxiety; Constant vs No Anxiety; High vs Moderate Anxiety; High vs Mild Anxiety; High vs No Anxiety; Moderate vs Mild Anxiety; Moderate vs No Anxiety; Mild vs No Anxiety.
