# Supplementary material for: Comparison of embryologist stress, somatization, and burnout reported by embryologists working in UK HFEA-licensed ART/IVF clinics and USA ART/IVF clinics
Source: Hum Reprod. 2024 Aug 28;39(10):2297–304. doi: 10.1093/humrep/deae191 (PMC11447060; doi:10.1093/humrep/deae191)
Supplement: deae191_Supplementary_Figure_S18 [file deae191_supplementary_figure_s18.pdf]

Human Reproduction, 2024, 1–1
https://doi.org/10.1093/humrep/deae191
Supplementary Data

Level of Anxiety re: Cryostorage	People		PSS		PHQ-15	
	#	%	Score	STD	Score	STD
Constant Anxiety	22	9%	19.41	6.34	11.91	5.54
High Anxiety	50	20%	18.14	5.58	11.66	4.14
Moderate Anxiety	73	30%	18.93	5.46	7.95	5.12
Mild Anxiety	77	31%	16.04	4.79	10.29	4.76
No Anxiety	23	9%	13.48	5.24	4.00	NA
NA	1	0%	12.00	NA	6.09	5.29
**Grand Total**	**246**	**100%**	**16.33**	**5.48**	**8.65**	**4.97**

Supplementary Figure S18. Levels of anxiety re: Cryostorage, PSS, and PHQ-15 in the US.

PSS and PHQ-15 of working conditions with a statistically significant difference: $P < 0.05$.

PSS: Constant vs Mild Anxiety; Constant vs No Anxiety; High vs Mild Anxiety; High vs No Anxiety; Moderate vs Mild Anxiety; Moderate vs No Anxiety; and Mild vs No Anxiety.

PHQ-15: Constant vs Mild Anxiety; Constant vs No Anxiety; High vs Mild Anxiety; High vs No Anxiety; Moderate vs Mild Anxiety; and Moderate vs No Anxiety.

Color coding: PSS: Red—high, yellow—moderate, and light-green—low; PHQ-15: burgundy—high, deep-yellow—medium, green—low, and deep-green—minimal.
