# Supplementary material for: Comparison of embryologist stress, somatization, and burnout reported by embryologists working in UK HFEA-licensed ART/IVF clinics and USA ART/IVF clinics
Source: Hum Reprod. 2024 Aug 28;39(10):2297–304. doi: 10.1093/humrep/deae191 (PMC11447060; doi:10.1093/humrep/deae191)
Supplement: deae191_Supplementary_Table_S1 [file deae191_supplementary_table_s1.pdf]

OXFORD
human reproduction
Human Reproduction, 2024, 1–1
https://doi.org/10.1093/humrep/deae191
Supplementary Data

Supplementary Table S1. Adjusted* change and 95% CI in Perceived Stress Scale (PSS) and Patient Health Questionnaire (PHQ)-15 scores associated with employment characteristics.

	UK EFS		US EFS	
**Characteristic**	**PSS change (95% CI)**	**PHQ-15 change (95% CI)**	**PSS change (95% CI)**	**PHQ-15 change (95% CI)**
**Inadequate vs adequate staffing**	**4.66 (2.42–6.90)**	**1.96 (0.17–3.76)**	**3.09 (1.47–4.72)**	**2.34 (0.83–3.85)**
Present vs absent anxiety re: being on-call	2.34 (−0.01 to 4.69)	1.31 (−0.49 to 3.12)	**2.51 (1.06–3.95)**	1.16 (−0.18 to 2.51)
Mandatory vs voluntary overtime	0.35 (−2.25 to 2.95)	0.41 (−1.57 to 2.40)	0.96 (−0.51 to 2.42)	−0.39 (−1.74 to 0.97)
Inflexible vs flexible scheduling	0.33 (−2.12 to 2.77)	1.29 (−0.81 to 3.39)	**2.93 (1.55–4.30)**	**1.59 (0.30–2.88)**
**Ever vs never missing life events due to work**	**4.08 (1.79–6.38)**	**2.85 (1.08–4.61)**	**3.71 (2.02–5.41)**	**3.36 (1.80–4.92)**

* Adjusted for years as an embryologist, full-time vs part-time/per-diem working status, and doctorate-level education. Bold denotes a statistically significant dose-dependent effect of employment characteristics on PSS and PHQ-15 changes.
