# Supplementary material for: Comparison of embryologist stress, somatization, and burnout reported by embryologists working in UK HFEA-licensed ART/IVF clinics and USA ART/IVF clinics
Source: Hum Reprod. 2024 Aug 28;39(10):2297–304. doi: 10.1093/humrep/deae191 (PMC11447060; doi:10.1093/humrep/deae191)
Supplement: deae191_Supplementary_Table_S2 [file deae191_supplementary_table_s2.pdf]

OXFORD human reproduction

Human Reproduction, 2024, 1–1
https://doi.org/10.1093/humrep/deae191
Supplementary Data

Supplementary Table S2. Unadjusted and adjusted risk ratios (RR) and 95% confidence intervals (CI) of high levels of burnout in at least two domains associated with employment characteristics.

	UK EFS		US EFS	
**Characteristic**	**Unadjusted RR (95% CI)**	**Adjusted* aRR (95% CI)**	**Unadjusted RR (95% CI)**	**Adjusted* aRR (95% CI)**
Inadequate vs adequate on-call staffing	1.20 (0.89–1.62)	1.22 (0.88–1.70)	1.55 (1.15–2.09)	**1.63 (1.22–2.17)**
Present vs absent anxiety re: being on-call	1.61 (1.19–2.19)	**1.62 (1.13–2.31)**	1.22 (1.02–1.45)	**1.32 (1.13–1.54)**
Overtime vs no overtime	1.89 (1.06–3.40)	**1.82 (1.01–3.29)**	1.26 (0.96–1.65)	1.13 (0.88–1.46)
Mandatory vs voluntary overtime	1.18 (0.87–1.59)	1.22 (0.89–1.68)	1.39 (1.16–1.68)	**1.22 (1.02–1.47)**
Uncompensated vs compensated overtime	1.38 (1.04–1.84)	1.36 (0.99–1.87)	1.19 (0.98–1.45)	**1.17 (0.97–1.41)**
Inadequate vs adequate eve/wknd compensation	1.50 (1.05–2.15)	**1.64 (1.04–2.61)**	1.15 (0.88–1.51)	**1.23 (0.95–1.59)**
Inability vs ability to take two consecutive days off	1.21 (0.90–1.63)	1.21 (0.88–1.66)	1.07 (0.88–1.29)	1.12 (0.94–1.34)
Inflexible vs flexible scheduling	1.50 (1.10–2.05)	**1.49 (1.09–2.04)**	1.13 (0.94–1.36)	**1.21 (1.02–1.43)**
Ever vs never missing key life events due to work	2.02 (1.40–2.91)	**1.97 (1.36–2.85)**	1.52 (1.11–2.08)	**1.58 (1.17–2.14)**

* Adjusted for years as an embryologist, full-time vs part-time/per-diem working status, and doctorate-level education. Bold denotes a statistically significant dose-dependent effect of employment characteristics on burnout in adjusted analyses.

© The Author(s) 2024. Published by Oxford University Press on behalf of European Society of Human Reproduction and Embryology.
This is an Open Access article distributed under the terms of the Creative Commons Attribution License (https://creativecommons.org/licenses/by/4.0/), which permits unrestricted reuse, distribution, and reproduction in any medium, provided the original work is properly cited.
Human Reproduction, 2024, 00, 1–9
https://doi.org/10.1093/humrep/deae191
Original Article
